# Rare sex or out of reach equilibrium? The dynamics of *F*_*IS*_ in partially clonal organisms

**DOI:** 10.1186/s12863-016-0388-z

**Published:** 2016-06-10

**Authors:** Katja Reichel, Jean-Pierre Masson, Florent Malrieu, Sophie Arnaud-Haond, Solenn Stoeckel

**Affiliations:** IGEPP, Agrocampus Ouest, INRA, Université de Rennes 1, 35650 Le Rheu, France; Université de Tours, CNRS-UMR7350 LMPT, F-37200 Tours, France; IFREMER, UMR5240 MARBEC, F-34203 Sète, France

**Keywords:** Partial asexuality, Parthenogenesis, Mating system, Inbreeding coefficient, Heterozygote excess, Genetic diversity

## Abstract

**Background:**

Partially clonal organisms are very common in nature, yet the influence of partial asexuality on the temporal dynamics of genetic diversity remains poorly understood. Mathematical models accounting for clonality predict deviations only for extremely rare sex and only towards mean inbreeding coefficient $\overline{F_{IS}}<0$. Yet in partially clonal species, both *F*_*IS*_ < 0 and *F*_*IS*_ > 0 are frequently observed also in populations where there is evidence for a significant amount of sexual reproduction. Here, we studied the joint effects of partial clonality, mutation and genetic drift with a state-and-time discrete Markov chain model to describe the dynamics of *F*_*IS*_ over time under increasing rates of clonality.

**Results:**

Results of the mathematical model and simulations show that partial clonality slows down the asymptotic convergence to *F*_*IS*_ = 0. Thus, although clonality alone does not lead to departures from Hardy-Weinberg expectations once reached the final equilibrium state, both negative and positive *F*_*IS*_ values can arise transiently even at intermediate rates of clonality. More importantly, such “transient” departures from Hardy Weinberg proportions may last long as clonality tunes up the temporal variation of *F*_*IS*_ and reduces its rate of change over time, leading to a hyperbolic increase of the maximal time needed to reach the final mean $\overline{F_{IS,\infty }}$ value expected at equilibrium.

**Conclusion:**

Our results argue for a dynamical interpretation of *F*_*IS*_ in clonal populations. Negative values cannot be interpreted as unequivocal evidence for extremely scarce sex but also as intermediate rates of clonality in finite populations. Complementary observations (e.g. frequency distribution of multiloci genotypes, population history) or time series data may help to discriminate between different possible conclusions on the extent of clonality when mean $\overline{F_{IS}}$ values deviating from zero and/or a large variation of *F*_*IS*_ over loci are observed.

**Electronic supplementary material:**

The online version of this article (doi:10.1186/s12863-016-0388-z) contains supplementary material, which is available to authorized users.

## Background

Reproductive systems impact the evolution of genetic diversity at the population level [1, 2], making them an important factor for considerations on the evolvability of species. Partially clonal species, i.e. species that are able to reproduce both sexually and clonally, are common across many phyla and ecosystems [3] and represent an important part of the global biodiversity. They include many species of which evolutions are directly impacted by, or impacting humans, such as cultivated species [4], pathogens [5], invasive species [6], and species threatened by extinction (e.g. [7–11]). Partially clonal species are therefore frequently the subject of molecular analyses describing their genetic diversity [12], and the conclusions drawn depend on a correct understanding of the effects of their reproductive mode on the genetic composition of their populations.

The interpretation of standard population genetic indices from partially clonal populations can be challenging, as expectations are likely to depend on the rate of clonality, which is usually unknown in natural populations. The estimate of this rate on the basis of indirect approaches such as population genetics analysis remains elusive. One example of an index that has been suggested to change with the rate of clonality is *F*_*IS*_ [13, 14]. Within diploid individuals, it represents a correlation coefficient among alleles at a particular locus, and depends on their tendency to be randomly associated *F*_*IS*_ = 0 or more likely identical (*F*_*IS*_ > 0) or not identical (*F*_*IS*_ < 0) at the population level. *F*_*IS*_is defined either based on population heterozygosity (*H*_*e*_ – expected heterozygosity, *H*_*o*_ – observed heterozygosity) or allele identities/homozygosity (*F* – allele identity within individuals, *Θ* – allele identity within the population [13]):

$$ {F}_{IS}=\frac{H_e-{H}_o}{H_e}\cong \frac{F-\Theta}{1-\Theta}, \kern0.5em {F}_{IS}\in \left[-1,1\right] $$

Results from both definitions differ only for loci with just a single allele remaining (fixation), where *F*_*IS*_ cannot be defined.

To date, only few mathematical models studying *F*_*IS*_ at selectively neutral loci in partially clonal populations have been published. For partially clonal populations otherwise complying with the Hardy-Weinberg conditions, *F*_*IS*_ and the underlying genotype frequencies are expected to be identical to those expected for random mating, except for the approach to the Hardy-Weinberg equilibrium (HWE) being slowed down as the rate of clonality increases [15]. If mutation and genetic drift are taken into account [13], very high rates of sexual reproduction are expected to lead eventually to strongly negative mean *F*_*IS*_ values up to $\overline{F_{IS,\infty }}=-1$ for completely clonal populations. In addition to this effect on the mean, the shape of the full steady state (i.e. “equilibrium”: the convergence of values toward a given probabilistic and dynamically-stable distribution) distribution of *F*_*IS*_, measured by its variance, skewness and kurtosis, also changes with the rate of clonality [16]. Based on the results of [13], $\overline{F_{IS}}$ was suggested as an informative parameter to estimate the rate of clonality [14, 17] in connection with other indices such as linkage disequilibrium or the frequency of repeated multiloci genotypes [18]. However, using the mean of the steady state distribution provided by [13] as a reference for the mean $\overline{F_{IS}}$ values from field studies often pointed to rates of clonality that were at odds with other indices or even direct observation (e.g. [19, 20]).

While some previous works may be interpreted as demonstrating negative *F*_*IS*_ as a signature of nearly exclusive clonality [13, 14], others underline the influence of clonality not only on the steady state distributions of *F*_*IS*_ but also on the temporal dynamics of this parameter in natural population [15, 16]. Here we aimed at complementing the results of the previous studies by describing the temporal changes of genotype frequencies over time under the distinct and joint influence of partial clonality, mutation and genetic drift. Understanding how quickly the steady state distribution of *F*_*IS*_ is reached again after a disturbance (e.g. change of reproductive system, stochastic or selective events) due to clonality, mutation and drift, may indeed help to explain the unexpected departures observed in nature, in populations otherwise considered as undergoing rather frequent sexual reproduction.

We used a stochastic model to follow the neutral dynamics of genotype frequncies in the basic case of a single locus in a diploid, isolated and panmictic population that combines random mating and clonality. We derived the dynamical effects of population size, mutation rate and rate of clonality separately on genotype frequencies over generations and how long it takes until a steady state distribution is reached again after any disturbance. We subsequently connected these partial results to analyze the “complete” system with the joint effects of reproductive mode, mutation and genetic drift. Finally, we discuss how our results may provide new hypotheses in the interpretation of field data, based on examples from a literature review, and we provide methodological recommendations for future analyses of genotype frequencies in partially clonal populations.

## Methods

### Mathematical model

**Fig. 1:**
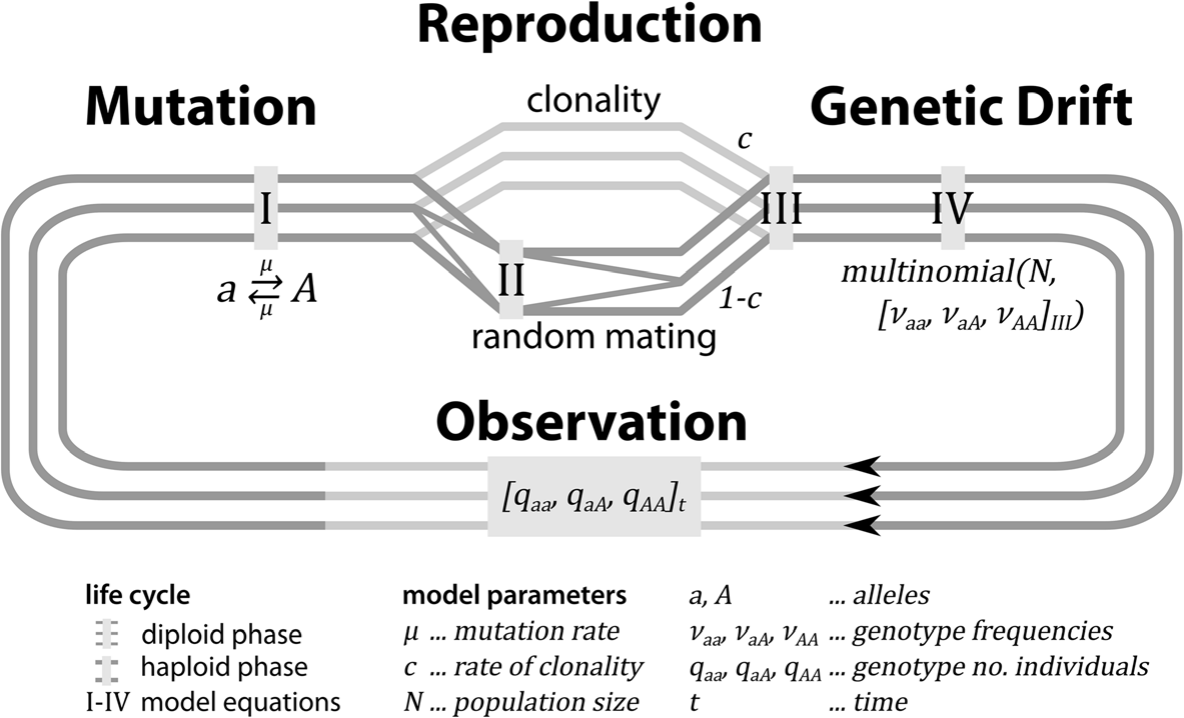
Schematic overview of the mathematical model (example for two alleles). In a dominantly diploid population of fixed size *N*, the number of individuals/ramets *q* with a certain genotype (here *aa, aA,* or *AA*) at a particular locus, observed at generation *t*, and the corresponding genotype frequencies *ν* = *q*/*N* may change due to mutation (here symmetrical from *a* to *A* and from *A* to *a* with rate *μ*; see Eq. 1), reproduction (random mating at rate 1 − *c*; see Eqs. 2 and 3) or genetic drift (modeled by multinomial drawing of *N* individuals from the genotype frequency distribution; see Eq. 4), until observation at the next generation

The biological template for our model is a single population with a finite number of individuals. These individuals correspond to ramets, i.e. factually or potentially physiologically distinct units that may or may not be genetically identical or descended from the same parent. All individuals follow the same life cycle, which consists of a dominant diploid phase during which they can acquire heritable mutations (Fig. 1). All individuals are monoecious and can produce offspring both by clonal and sexual (here defined as random mating including selfing) reproduction. A fixed number of these offspring individuals corresponding to a constant population size survive randomly to replace their parents in the next generation.

We translated this system into a time and state discrete Markov chain model, conceptually similar to [16] to follow the population dynamics of genotype frequencies at one locus with multiple alleles, as in the classic models of Wright [21] and others [22, 23]. Each time step of the model corresponds to one generation, i.e. the time between two consecutive observations of the population (Fig. 1). The model states represent all possible distributions of the *N* individuals on *g* genotypes (a genotypic state): For a single locus *n* alleles, the number of genotypes corresponds to *g* = *n*(*n* + 1)/2 and the number of states to (*N* + *g* − 1) !/(*N* ! ⋅ (*g* − 1) !). For example, a locus of 3 alleles (A1, A2 and A3) results in 6 possible genotypes (A1A1, A1A2, A1A3, A2A2, A2A3, A3A3) and a population composed of 100 individuals could evolve among the 96 560 646 single possible repartitions of 100 individuals within the 6 genotypes (hereafter named a population state) [16, 24].

At each time step, the population makes a transition from its current state to a next state (where current and next state can be the same), based on a vector of transition probabilities. These probabilities depend on the genotype frequencies *ν*_*ii*_, *ν*_*ij*_ (with the indices *i* ≠ *j* denoting different alleles) derived from the current population state, and on the three constant model parameters: population size *N*, mutation rate *μ* and rate of clonality *c*, according to the following equations (Fig. 1; simplified for the special case of two alleles in Additional file 1, 1.1):

**I Mutation.** The theoretical frequencies *ν*_*ii*,*I*_, *ν*_*ij*,*I*_ of each genotype after mutation are derived as:

1 $$ \left\{\begin{array}{c}\hfill {\nu}_{ii,I}={\alpha}^2{\nu}_{ii,\ t}+\alpha \beta {\displaystyle {\sum}_{j\ne i}{\nu}_{ij,t}} + {\beta}^2\left({\displaystyle {\sum}_{j\ne i}{\nu}_{jj,t}}+{\displaystyle {\sum}_{k,j\ne i}{\nu}_{jk,t}}\right)\hfill \\ {}\hfill {\nu}_{ij,I}=\left({\alpha}^2+{\beta}^2\right){\nu}_{ij,\ t}+2\alpha \beta \left({\nu}_{ii,\ t}+{\nu}_{jj,\ t}\right) + \left(\alpha \beta +{\beta}^2\right)\left({\displaystyle {\sum}_{k\ne i,j}{\nu}_{ik,t}}+{\displaystyle {\sum}_{l\ne i,j}{\nu}_{jl,t}}\right)+2{\beta}^2\left({\displaystyle {\sum}_{k\ne i,j}{\nu}_{kk,t}}+{\displaystyle {\sum}_{k,l\ne i,j}{\nu}_{kl,t}}\right)\hfill \end{array}\right. $$

where *α* = 1 − *μ*, the probability that an allele does not mutate, and $\beta =\frac{\mu }{n-1}$, the probability that an allele mutates into one of the *n* − 1 others during one generation. This corresponds to a classic k-alleles or Jukes-Cantor substitution model [25].

**II Gamete formation (allele segregation then fusion).** The gamete frequencies in the gamete pool after sexual reproduction *ν*_*i*,*I*_ are calculated as:

2 $$ {\nu}_{i,I}={\nu}_{ii,I}+\frac{1}{2}{\displaystyle {\sum}_{j\ne i}{\nu}_{ij,I}} $$

There is no difference in the allele frequencies between sexes, mating types etc., and all individuals contribute equally to the gamete pool (pangamy).

**III Reproduction.** The genotype frequencies *ν*_*ii*,*III*_, *ν*_*ij*,*III*_after reproduction are calculated as:

3 $$ \left\{\begin{array}{c}\hfill {\nu}_{ii,III}=c{\nu}_{ii,I}+\left(1-c\right){\nu_{i,I}}^2\hfill \\ {}\hfill {\nu}_{ij,III}=c{\nu}_{ij,I}+2\left(1-c\right){\nu}_{i,I}{\nu}_{j,I}\hfill \end{array}\right. $$

based on the results from Eqs. 1 and 2. The rate of clonality *c* thus corresponds to the proportion of offspring per generation that is the result of clonal reproduction. The remainder of the offspring (“rate of sexuality”, 1 − *c*) is derived from random mating including autogamy, assuming panmixis.

**IV Genetic Drift.** The vector of genotype frequencies ${\overrightarrow{v}}_{t+1}$ at the next generation depending on the population size is derived from:

4 $$ {\overrightarrow{\nu}}_{t+1}=\frac{X_{t+1}}{N}\ \mathrm{where}\ {X}_{t+1} \sim \mathrm{\mathcal{M}}\left(N,\ {\overrightarrow{\nu}}_{III}\right) $$

where *X*_*t*+1_ is the state of the model at the next generation, drawn from a multinomial distribution *M* that is based on *N*, the population size counting all potentially reproducing individuals (mathematically the number of samples), and ${\overrightarrow{\nu}}_{III}$, the vector of genotype frequencies derived from Eq. 3 (mathematically the probabilities of the genotype “categories”). Transition probabilities *P* between any two model states *X*_*t*_, *X*_*t* + 1_ can then be calculated based on:

5 $$ P\left({X}_{t+1}\Big|{X}_t\right)=\frac{N!}{{\displaystyle {\prod}_i}{q}_{ii,\ t+1}!{\displaystyle {\prod}_{i,j}}{q}_{ij,\ t+1}!}{\displaystyle {\prod}_i{\nu}_{ii,III}^{q_{ii,\ t+1}}{\displaystyle {\prod}_{i,j}{\nu}_{ij,III}^{q_{ij,\ t+1}}}} $$

where *q*_*ii*, *t* + 1_, *q*_*ij*, *t* + 1_ ∈ ℕ_0_ are the counts (natural numbers) of individuals per genotype in the presumed next state *X*_*t*+1_ which sum to *N*. Note that our description of genetic drift is based on genotype frequencies rather than allele frequencies. As explained in [22], describing population genetic processes based on allele frequencies is a mathematical convenience justified by HWE (i.e. assuming exclusively sexual reproduction), which assures that allele frequencies can always be directly translated into genotype frequencies. For partially clonal populations, we cannot automatically assume HWE and thus modeled all population genetic processes, including genetic drift, at the genotypic level.

### Model analysis and description of biological consequences

First, we studied the effect of each of the three model parameters (*c*, *μ*, *N*) on the genotype frequencies by itself. Setting the other two parameters to have no influence on the model result, i.e. *c* = 1, *μ* = 0 and/or *N* = ∞ (or no random drawing in Eq. 4), and substituting Eq. 2 into Eq. 3, the model reduces to one equation per process, i.e. Eq. 1 for *μ*, Eq. 3 (with 2) for *c*, and Eq. 4 for *N*. For each equation/process, we then determined the steady state distributions of genotype frequencies at one locus, i.e. the probabilistic combination of population states for which ${\overrightarrow{q}}_{t+1}={\overrightarrow{q}}_t$. We also derived the respective maximal convergence times *t*_*c*_, *t*_*μ*_ and *t*_*N*_ of each separate evolutionary process to reach steady state distributions of genotype frequencies. While *t*_*N*_ could only be approximated from numerical results (Markov chain first passage time approach and simulations), for *c* and *μ* convergence to the steady states is asymptotical as it can be described by geometric progressions (details of derivation in Additional file 1, 1.2). We therefore defined a universal “acceptable error” *ɛ* = 1/(2*N*), corresponding to one half the minimal change in genotype frequency that would be measurable by exhaustive sampling in a population of finite size *N*, below which the distance from the steady states has to pass (convergence criterion). Using the maximal convergence times *t*_*c*_, *t*_*μ*_ and *t*_*N*_ as measures for the “strength” with which each process acts upon the genotype frequencies, we could then use this analytical basis to partition the parameter space of the full model into regions where either process dominates the genotype frequency dynamics.

Secondly, we approached the full model by following the dynamics of *F*_*IS*_ over time from three different start states for combinations of *c*, *μ* and *N* representative of the different regions of the parameter space. Aggregating the transition probabilities between all model states in a transition matrix *M* (same current state per column, i.e. columns summing to one), the probability distribution of the model states (and consequently the probability distribution of *F*_*IS*_) at time *t*, given by the vector ${\overrightarrow{x}}_t$, is derived by matrix multiplication:

6 $$ {\overrightarrow{x}}_t={M}^t{\overrightarrow{x}}_0 $$

where ${\overrightarrow{x}}_0$ describes the start state (vector of zeros except for a single one at *X*_0_). The steady state distribution of population states corresponds to the dominant eigenvector of the transition matrix *M*. Based on the transition matrix *M*, we also calculated the time to the steady state distribution (Markov chain mixing time, Additional file 1, 1.5).

We illustrated the numerical result of our model using three start states: *F*_*IS*,0_ ∊ { − 1; 0; 1} under isoplethic allele frequencies (equally frequent, ${\nu}_a={\nu}_A=\frac{1}{n}$), standing for HWE (*F*_*IS*,0_ = 0) and the most extreme deviations from it (complete homozygosity, *F*_*IS*,0_ = 1; complete heterozygosity, *F*_*IS*,0_ = − 1). These start states were chosen to represent the range of *F*_*IS*_ values, and not because of their biological meaning or how they may be reached in nature. Deviations from steady state distributions may derive from a recent change in the rate of clonality (e.g. from full sexuality with *F*_*IS*,0_ = 0), or full adaptation to past selection for (*F*_*IS*,0_ = − 1) or against (*F*_*IS*,0_ = 1) heterozygotes, or changes in population size (demographic bottleneck, founder event) [26, 27], or secondary contact between two populations in which different alleles become fixed (*F*_*IS*,0_ = 1) or hybridization with subsequent reproductive isolation from the parents (*F*_*IS*,0_ = − 1) (e.g. [28–30]). Based on the transition matrix *M*, we also calculated the time to the final distribution of states (= steady states), which is also the time until the exact final distribution of $\tilde{{F_{IS}}_{,\infty }}$ (Markov chain mixing time, Additional file 1, 1.5).

To link our results with those obtained by previous authors, we calculated the final mean $\overline{{F_{IS}}_{,\infty }}$ from Eq. 10 in [13] formalized for metapopulation:

$$ {F}_{IS}=\frac{\gamma \left({q}_s-c\left(\gamma \left({q}_s-{q}_d\right)-1\right)-1\right)}{2N\left(1-\gamma c\right)\left(\gamma \left({q}_s-{q}_d\right)-1\right)-\gamma \left({q}_s-c\left(\gamma \left({q}_s-{q}_d\right)-1\right)-1\right)} $$

where *γ* = (1 − *μ*)^2^, *q*_*s*_ is the probabilities that two individuals taken at random in the same reproductive subpopulation after migration were sired in the same reproductive subpopulation one generation before, and *q*_*d*_ the probability that two individuals taken at random in different reproductive subpopulations after migration originated from the same subpopulation one generation before.

By setting *q*_*s*_ = 1, *q*_*d*_ = 0 (a single finite population, no migration) and *s* = 1/*N* (random mating):

7 $$ \overline{F_{IS\infty }}=\frac{1}{\left(2N-1\right)-2N/c{\left(1-\mu \right)}^2} $$

We derived the corresponding expected convergence time of $\overline{F_{IS}}$ iteratively obtained from Eq. 5 in [13] (detailed in Additional file 1, 1.6)

8 $$ \left[\begin{array}{c}\hfill 1-{H}_{o,\kern0.5em t+1}\hfill \\ {}\hfill 1-{H}_{e,t+1}\hfill \end{array}\right]=\left[\begin{array}{c}\hfill {F}_{t+1}\hfill \\ {}\hfill {\Theta}_{t+1}\hfill \end{array}\right]={\left(1-\mu \right)}^2\left(\left[\begin{array}{cc}\hfill c+\frac{1-c}{2N}\hfill & \hfill \left(1-c\right)\left(1-\frac{1}{N}\right)\hfill \\ {}\hfill \frac{1}{2N}\hfill & \hfill 1-\frac{1}{N}\hfill \end{array}\right]\left[\begin{array}{c}\hfill {F}_t\hfill \\ {}\hfill {\Theta}_t\hfill \end{array}\right]+\left[\begin{array}{c}\hfill \frac{1-c}{N}\hfill \\ {}\hfill \frac{1}{2N}\hfill \end{array}\right]\right) $$

Where *F* and *θ* are the allele identities within individuals and between individuals respectively. In contrast to our own model, these equations do not contain the number of alleles. This is because they are based on an infinite alleles model, and treat the expected and observed hetero-/homo-zygosity as continuous variables whatever the population size considered.

Finally, to get a better idea how our theoretical results are comparable to those published for field data, we looked at the sampling effect of using different numbers of polymorphic loci *L* to estimate the mean $\overline{F_{IS}}$ of the population at time *t*, $\overline{F_{IS,t,L}}$. Assuming that each locus represents an independent estimate of this mean (no confounding effect of linkage), and that the genotype frequencies are known exactly (exhaustive sampling of all individuals/ramets), it is derived as:

9 $$ \overline{F_{IS,t,L}}=\frac{1}{L}{\displaystyle {\sum}_{z=0}^L{F}_{IS,t,z}} $$

Both assumptions are usually violated [14, 18], so that our results represent a conservative estimate of the true error of this method. We randomly sampled both the steady state distribution $\tilde{{F_{IS}}_{,\infty }}$ and the instantaneous distribution $\tilde{{F_{IS}}_{,50}}$ of a population that was 50 generations ago at HWE at all loci with equal allele frequencies (isoplethy for two alleles per locus), for the same parameter combinations that we previously used to illustrate the dynamics of the full model. Based on 10^5^ random samples of size *L*, we then calculated the mean signed deviation of the sample means *F*_*IS*,*t*_^⋅^ from the true mean $\overline{F_{IS,t}}$:

10 $$ \varDelta \overline{F_{IS,t}}=\left\{\begin{array}{c}\hfill \frac{1}{z_1}{\displaystyle {\sum}_{F_{IS,t}^{\cdot}\ge \overline{F_{IS,t}}}{F}_{IS,t}^{\cdot }}-\overline{F_{IS,t}}\hfill \\ {}\hfill \frac{1}{z_2}{\displaystyle {\sum}_{F_{IS,t}^{\cdot }<\overline{F_{IS,t}}}{F}_{IS,t}^{\cdot }}-\overline{F_{IS,t}}\hfill \end{array}\right., \kern0.5em {z}_1+{z}_2={10}^5 $$

where *z*_1_ and *z*_2_ represent the number of positive and negative deviations, respectively. Loci at or near fixation are typically not used in population genetic studies, since they are especially affected by genotyping errors. We therefore excluded all loci where the frequency of one allele exceeds $1-\sqrt{1/(2N)}$ (near fixation; Additional file 2: Figure S2.1 for the derivation of this value, and compare similar considerations in [31]) from the calculation of values for this analysis.

**Fig. 2:**
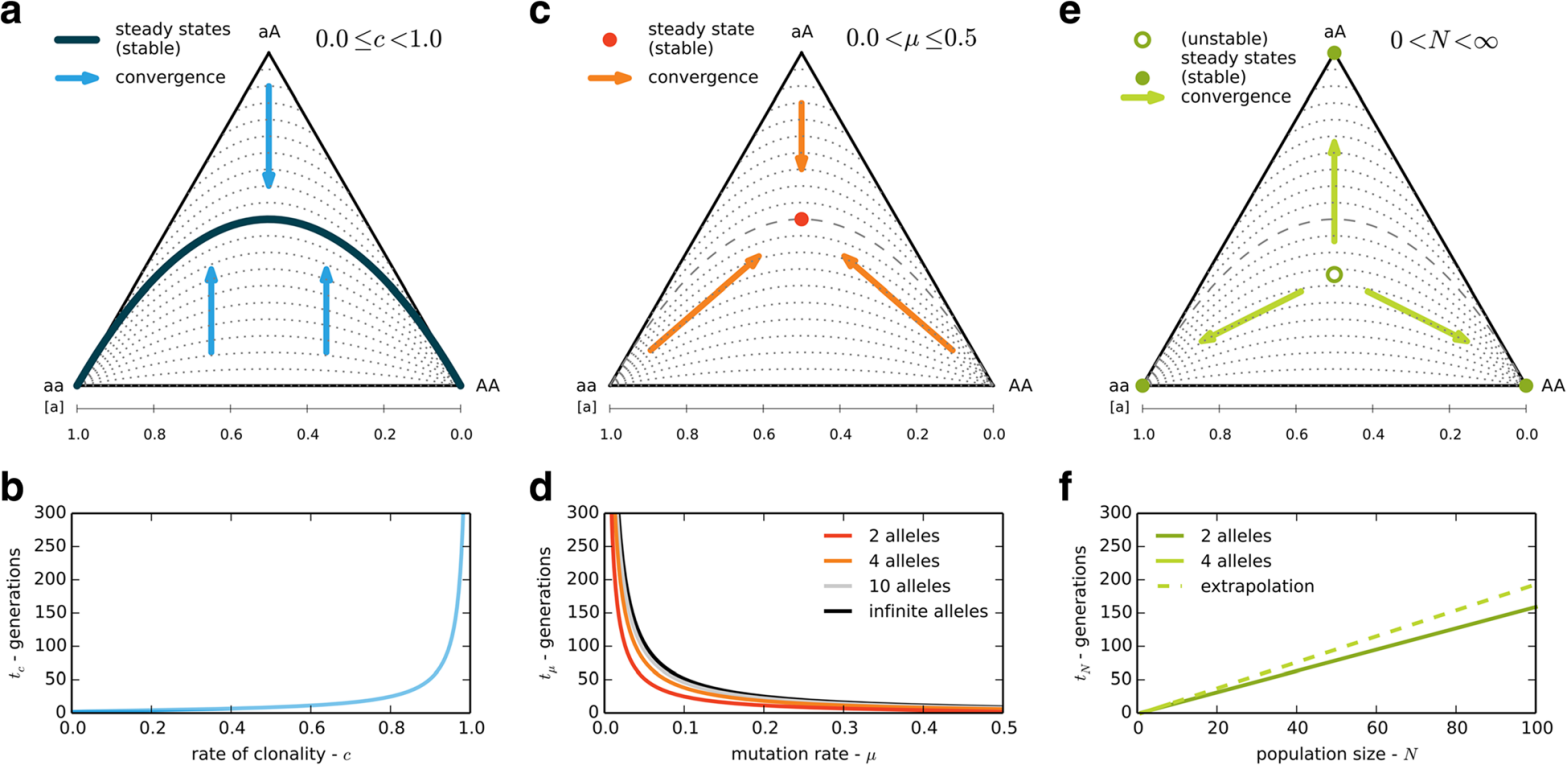
Genotype dynamics due to individual model parameters only. Discontinuous grey lines connect states of equal *F*_*IS*_ (dashed: *F*_*IS*_ = 0, dotted: *F*_*IS*_ = ± 0.1, 0.2 … 1) Arrows indicate the direction of genotype frequency change over time, filled colored dots/line the stable steady states where genotype frequencies do not change anymore, and unfilled colored dots the unstable steady states where genotype frequencies do not change in the mean. **a** Reproduction convergence pattern (random mating + clonality) for 0.0 ≤ *c* < 1.0, based on Additional file 2: Figure S2.2. No genotype frequency changes due to reproduction for *c* = 1.0. **b** Maximal expected convergence time *t*_*c*_ in generations for each rate of clonality *c*. **c** Mutation convergence pattern (k-alleles/Jukes-Cantor model) for 0.0 < *µ* ≤ 0.5, based on in Additional file 2: Figure S2.3. No genotype frequency changes due to mutation for *μ* = 0.0. **d** Maximal expected convergence time *t*_*μ*_ in generations for each rate of mutation *μ* and different numbers of alleles (red: 2, orange: 4, grey: 10, black: infinite). **e** Genetic drift convergence pattern for 0.0 < *N* < ∞, based on Additional file 2: Figure S2.4. No genotype frequency changes due to genetic drift for *N* = ∞. **f** Maximal expected convergence time *t*_*N*_ in generations for population sizes from 1 to 100, for two different numbers of alleles (darker green: 2, lighter green: 4). Results for four alleles are in part based on an extrapolation (dashed line) from numerical solutions for smaller population sizes

All computations were performed in Python 2.7 with 64 bit precision, using the modules *numpy, scipy* [32], *networkx* [33] and *matplotlib* [34] (basic code in Additional file 3). We illustrate some of our results with *de Finetti* diagrams [24, 35] (Fig. 2a, c, e), which are ternary plots of the genotype frequencies [*ν*_*aa*_, *ν*_*aA*_, *ν*_*AA*_] at one locus with two alleles within a population (see Additional file 2: Figures S2.1 to S2.4 for more information). Details for the literature review in the discussion are given in Additional file 4.

### Examples for empirical *F*_*IS*_ values of partially clonal populations from published field studies

To get a summary view of the kind of *F*_*IS*_ values that can be encountered in real field populations suspected to reproduce using partial clonality, we compiled the data for 51 populations belonging to 13 species (seven angiosperms, four protists, a sponge and a nematode), based on 13 previously published studies (see references in Additional file 3) selected for their near fit with the assumptions of our model from a Web of Science search for [(microsatellite *OR* “SSR” *OR* “simple sequence repeat” *OR* “SNP” *OR* “single nucleotide polymorphism”) *AND* (clonal *OR* asexual *OR* vegetative *OR* apomictic *OR* apomixis *OR* agamospermy *OR* parthenogenesis)]. All studies are based on SSR data. When *F*_*IS*_ values were not directly provided by publications, we calculated *F*_*IS*_ values per locus from the reported *H*_*o*_ and *H*_*e*_. We grouped populations into three classes according to the information given by the authors about their putative rate of clonality: *i)* rarely clonal, *ii)*frequent clonality and sexuality (including unknown) and *iii)*rarely sexual. We included populations for which preferential outbreeding (self-incompatibility system) was expected.

## Results

The maximal times until the genotypic diversity at one locus has converged to its steady state distribution changed greatly with the rates of clonality. During this convergence, the steady state distributions, the dynamics of *F*_*IS*_ and the underlying genotype frequencies through time were also affected by the rate of clonality. However, both maximal convergence times and the dynamics of genotypic diversity at one locus strongly depend on the interactions of the rate of clonality with mutation rate and population size. Indeed, each of these evolutionary processes has its own maximal convergence time toward the steady state distribution of genotype frequencies and *F*_*IS*_ (*t*_*c*_, *t*_*μ*_, *t*_*N*_). The overall convergence time of a population to its steady state distribution, which is shaped by mutation, genetic drift and the reproduction mode, depends on the relative “strength” of each of those three forces.

The maximal convergence time due to reproduction only, *t*_*c*_ (derived in Additional file 1, 1.2) can be approximated as

11 $$ {t}_c=1+{ \log}_c\varepsilon =1+\frac{ \log \varepsilon }{ \log c} $$

with $\varepsilon =\frac{1}{2N}$ corresponding to a small error term as convergence to the steady state distribution (HWE: Fig. 2a, Additional file 2: Figure S2.2) is asymptotic. Between *t*_*c*_ = 1 for exclusively sexual and *t*_*c*_ = ∞ for exclusively clonal populations, the dependence of *t*_*c*_ on *c* is not linear, but shapes like an hyperbola of type $y=1+\frac{constant}{x}$ (Fig. 2b). Consequently, the time required to reach a steady state distribution if only reproduction is considered increases hyperbolically as c increases.

The maximal convergence time due to mutation only *t*_*μ*_ (derived in Additional file 1, 1.2) can be approximated as

12 $$ {t}_{\mu }=1+{ \log}_{\left(1-\mu \frac{n}{n-1}\right)}\varepsilon =1+\frac{ \log \varepsilon }{ \log \left(1-\mu \frac{n}{n-1}\right),} $$

which simplifies for two alleles to

13 $$ {t}_{\mu }=1+{ \log}_{\left(1-2\mu \right)}\varepsilon =1+\frac{ \log \varepsilon }{ \log \left(1-2\mu \right)} $$

The steady state distribution for mutation corresponds to *F*_*IS*_ = 0 and, due to the symmetry of mutation between alleles in our models, equal allele frequencies (Additional file 1, 1.3; Additional file 2: Figure S2.3). For $\mu =\frac{1}{n}$, in theory the highest mutation rate (each allele has the same chance to mutate or not to mutate into other alleles), *t*_*μ*_ is only one generation. For realistic mutation rates ranging from 10^−3^ to 10^−18^ [36, 37], convergence due to mutation can take much longer: setting *ɛ* = 0.005 and assuming a mutation rate of *μ* = 10^−6^, *t*_*μ*_ would be around 2.6 ⋅ *μ*^−1^ or 2.6 million generations.

The maximal convergence time due to reach a steady state distribution (Fig. 2e, Additional file 2: Figure S2.4) due to genetic drift only *t*_*N*_ depends on the population size and on the number of initial genotypes (Fig. 2f). It grows approximately linearly with *N* and the slope of this dependence *σ* increases with the number of genotypes/alleles (Additional file 1, 1.2):

14 $$ {t}_N=\sigma N-\tau $$

with *τ* ≥ 1 and 1 < *σ* < 2 determined from numerical results (Additional file 2: Table S2.2) and simulations (Additional file 2: Table S2.3). For example, in a population of 100 individuals, up to about 160 generations can be required until only one genotype remains at a biallelic locus, and loci with more alleles take even a few generations more.

**Fig. 3:**
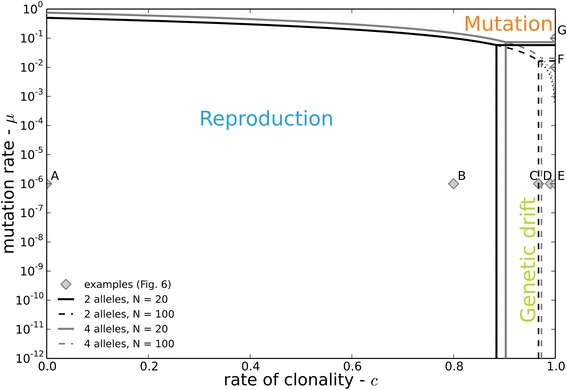
Overview of the model parameter space. With regions where the genotype dynamics are dominated by either reproduction, mutation or genetic drift. Lines correspond to *t*_*c*_ = *t*_*μ*_ *, t*_*c*_ = *t*_*N*_ *, t*_*μ*_ = *t*_*N*_ for two different numbers of alleles (black: 2 alleles, grey: 4 alleles) and two different population sizes (continuous: *N* = 20, dashed: *N* = 100). Labeled dots A-G indicate examples for which the dynamics of *F*_*IS*_ are shown in Fig. 4

The lowest maximal convergence time amongst *t*_*c*_, *t*_*μ*_, *t*_*N*_, can serve as approximation to determine the evolutionary process that dominates the dynamics of genotype frequencies and its associated steady state. Pairwise equality between the convergence times can thus be sued to partition the parameter space of our model distribution (eqs. 1 to 4, *c* ∈ [0, 1], *μ*∈]0, 0.5], *N* ∈ [1, ∞ [) into three domains where either process dominates (Fig. 3). Based on eqs. 12 to 14, these pairwise equalities resolve to:

15 $$ c=\mu \frac{n}{n-1} $$

16 $$ c={e}^{1/\left(\sigma N-\tau -1\right)} $$

17 $$ \mu =\frac{n-1}{n}\left(1-{e}^{1/\left(\sigma N-\tau -1\right)}\right) $$

If *t*_*c*_ ≪ *t*_*μ*_, *t*_*N*_, as is usually the case for strictly sexually reproducing populations, genotype frequencies quickly converge to HWE, i.e. *F*_*IS*,∞_ ≅ 0. In very large and highly clonal populations where , genotype frequencies also converge, although more slowly, to HWE so that *t*_*μ*_ ≪ *t*_*c*_*, t*_*N*_ is equally awaited in this case. This contrasts with small and highly clonal populations where *F*_*IS*,∞_ ≅ 0, as dominant genetic drift does not lead to convergence toward *t*_*N*_ ≪ *t*_*μ*_*, t*_*c*_, but rather to a successive loss of genotypes and eventually to genotypic uniformity (fixation of one homozygous or heterozygous genotype *F*_*IS*,∞_ = 0 or − 1). In conclusion, both random mating and mutation lead to a random association of alleles (Additional file 2: Table S2.1, [16]), so that the dynamics of *F*_*IS*_ in (partially) clonal populations is mostly driven by the relative “strength” of genetic drift.

A closer look at the transitions between the predominance of either process shows that changes are actually gradual. This is because different processes don’t globally compensate each other, as each convergence pattern is different (Fig. 2a, c, e, Additional file 2: Table S2.1). We therefore examined the joint action of all three processes in more details.

**Fig. 4:**
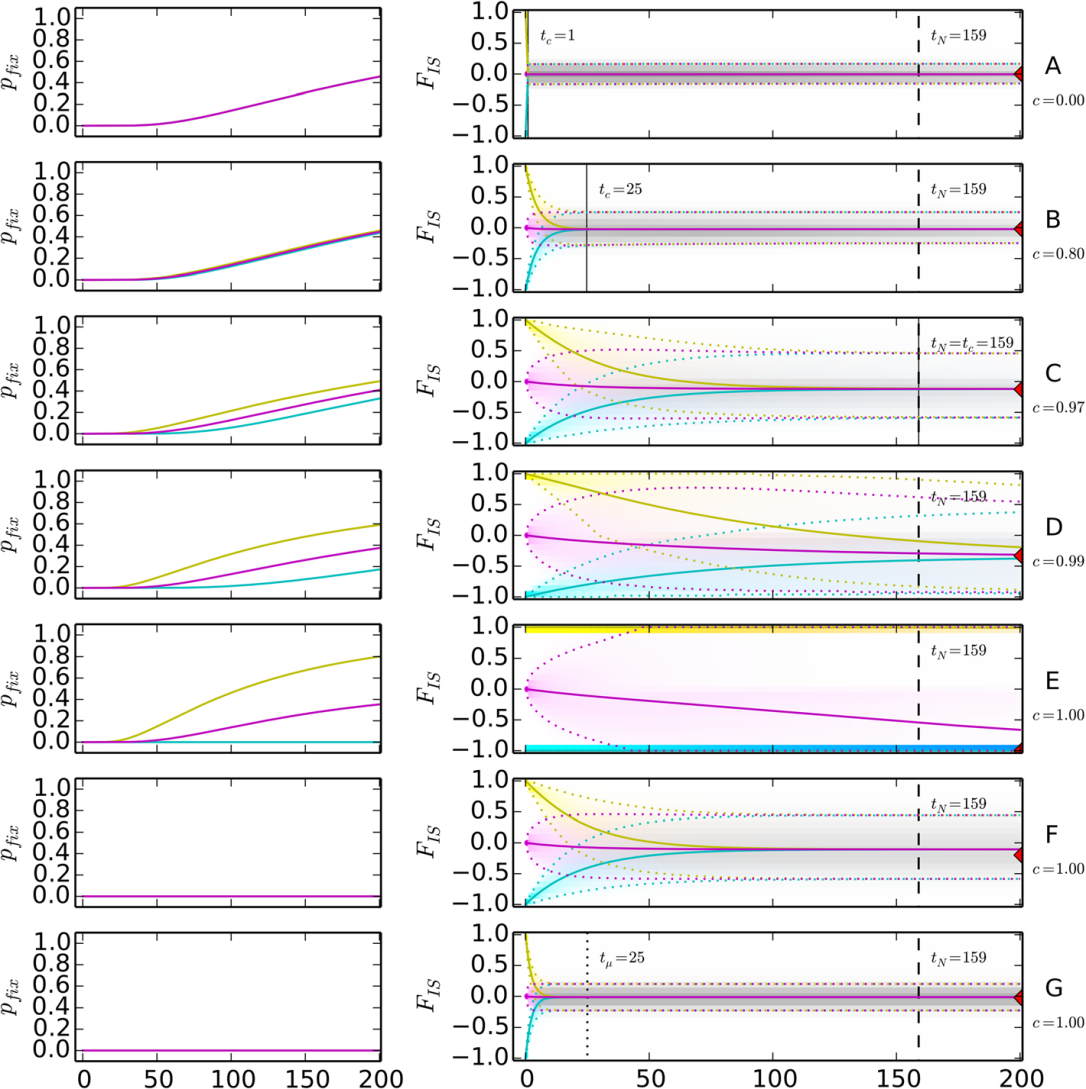
Dynamics of probability of fixation *p*_*fix*_ and *F*_*IS*_ through time for seven representative example parameter sets. Single loci with two alleles. Colors represent different start states (yellow: *F*_*IS*,0_ = 1 for *ν*_*a*_ = *ν*_*A*_, magenta: *F*_*IS*,0_ = 0 for *ν*_*a*_ = *ν*_*A*_, cyan: *F*_*IS*,0_ = − 1), with their respective $\overset{\sim }{F_{IS,t}}$ distributions (shading), mean (continuous line) and 95 % confidence intervall (dotted lines). Vertical lines represent *t*_*c*_ (continuous), *t*_*N*_ (dashed) and *t*_*μ*_ (dotted). Red triangles at *t* = 200 indicate the mean $\overline{F_{IS,\infty }}$ according to [13]. Model parameters – **a** *c* = 0, *μ* = 10^−6^, *N* = 100; **b** *c* = 0.8, *μ* = 10^−6^, *N* = 100; **c** *c* ≈ 0.97 (*t*_*c*_ = *t*_*N*_), *μ* = 10^−6^, *N* = 100; **d** *c* = 0.99, *μ* = 10^−6^, *N* = 100; **e** *c* = 1.0, *μ* = 10^−6^, *N* = 100; **f** *c* = 1.0, *μ* = 10^−2^, *N* = 100; **g** *c* = 1.0, *μ* = 10^−1^, *N* = 100

Keeping population size and mutation rate constant (*N* = 100, *μ* = 10^− 6^) while successively increasing the rate of clonality illustrates the changes in the dynamics of *F*_*IS*_ as genetic drift takes over (Fig. 4a to e, Additional file 2: Figures S2.6 and S2.7, Table S2.1). At low to intermediate rates of clonality (here 0 < *c* ≤ 0.8), the dynamics of *F*_*IS*_ appear almost identical to those expected for a purely sexual population (Fig. 4a and b). However, variation around the final mean $\overline{F_{IS,\infty }}$ is severely increased, and extreme initial *F*_*IS*,0_ values which would be lost in one generation under exclusive sexuality, can now be traced over a significant number of generations. These tendencies (increasingly negative $\overline{F_{IS,\infty }}$, increased variation of *F*_*IS*_ values, and increased time/start value dependence) continue until *t*_*c*_ crosses *t*_*N*_, leading to negative mean *F*_*IS*_ values ($c\approx 0.97:\ \overline{F_{IS,\infty }}\approx -0.14$, Fig. 4c) and then gain even further momentum as sexual reproduction becomes very rare, a situation in which *F*_*IS*_ reaches more negative mean *F*_*IS*_ values ($c=0.99:\ \overline{F_{IS,\infty }}\approx -0.33$, Fig. 4d). Indeed, transition probabilities that decrease *F*_*IS*_ raise while those that increase *F*_*IS*_ reduce (compare transition probabilities from (25,50,25) to (25,49,26) and to (25,51,24) in Additional file 2: Table S2.1). Moreover, probabilities to stay on the same genotypic state increase with clonality, except for states near fixation when mutation is high.

For loci starting with a high genotypic diversity, a marked difference in the positive and negative ranges of the *F*_*IS*_ distribution, namely the progressive disappearance of positive values, appears after some generations (*t* ~ 50, Fig. 4c-e). In parallel, the probability of allele fixation becomes increasingly dependent on the intial *F*_*IS*,0_ value (Fig. 4a-e, Additional file 2: Figures S2.6 and S2.7, left columns). The first reason for this effect, which also explains the convergence to negative $\overline{F_{IS,\infty }}$ in highly clonal populations, is the trend towards randomly increasing the frequency of the heterozygous genotype(s) at HWE and approximately evenly distributed allele frequencies (Additional file 1, 1.4). Indeed, HWE is a concave function in the *F*_*IS*_ space (for one locus, 2 alleles, see Additional file 2: Figure S2.1) which in probability facilitates slightly negative *F*_*IS*_ as a result of stochastic changes even for random mating populations (see Additional file 2: Figure S2.1 and Table S2.1, e.g. transition probabilities from (25,50,25) to (25,49,26) and (25,51,24)). Partial and full clonality only prevents populations, partly or completely, to get back to HWE, thus to express this feature over more generations. The second reason is the dependence of the rate of fixation (of one allele) on the initial *F*_*IS*,0_ at extreme clonal rate (Fig. 4a-e). All loci reaching positive *F*_*IS*,*t*_ values then have a higher chance to move quickly toward fixation, helped by each random mating event (as the HWE parabola in *de Finetti* diagrams connects fixation states) and thus to be usually excluded from *F*_*IS*_ calculation. Over time, this only leaves analyzable loci with negative values. It can be noted that contrastingly, loci shifting to negative *F*_*IS*,*t*_ (higher probabilities than shifting to positive, due to the concave shape of HWE) are unlikely to reach $\overline{F_{IS,\infty }}=-1.0$ by pure stochastic genetic drift, as each sexual and homoplasy events bring back populations toward the HWE concave parabola (Fig. 2a, c, e). Interestingly in our example, the mean $\overline{F_{IS,t}}$ (starting from *F*_*IS*,0_ = 0 in sexual populations) has not reached the final $\overline{F_{IS,\infty }}$ even after 200 generations while *t*_*N*_ = 159 because convergence to drift steady states is slowed down by “weak” mutation.

Highly clonal populations dominated by mutation rather than genetic drift present a different picture (Fig. 4f, g: *c* = 1.0, *μ* = {10^− 2^, 10^− 1^}, *N* = 100, or simulation for *c* = 1.0, *μ* = 10^− 3^, *N* = 10^3^ in Additional file 2: Figure S2.5). As in predominantly sexually reproducing populations, *F*_*IS*_ values converge to only slightly negative final $\overline{F_{IS,\infty }}$ (Fig. 4g: $\overline{F_{IS,\infty }}\approx -0.02$) but at a speed that mainly depends on *t*_*μ*_ and with a limited variation of *F*_*IS*_ values across generations. In contrast, the instantaneous $\overline{F_{IS,t}}$ distributions appears more symmetrical as the fixation of single alleles is very rare (Fig. 4f, g). As the maximal allelic diversity (number of possible alleles) increases beyond two, *t*_*μ*_ increases accordingly and mutation is “weakened” even further in comparison to the other processes (Fig. 2d).

The time until the exact final $\tilde{{F_{IS}}_{,\infty }}$ distribution is reached depends on the lowest force acting in the population. In realistic biological conditions, it typically depends on *t*_*μ*_ (Table 1, Additional file 1, 1.5). However, the mean $\overline{F_{IS,\infty }}$ may be reached much earlier than the time needed to reach the exact final $\tilde{{F_{IS}}_{,\infty }}$ distribution (Fig. 4, Table 1). Indeed, reaching the final mean $\overline{F_{IS,\infty }}$ only requires that populations have reached the final heterozygosity which depends on mutation and sexual reproduction. This final heterozygosity is quickly reached with few sexual events. Reaching the exact final $\tilde{{F_{IS}}_{,\infty }}$ distribution also implies having reached the steady state allele frequencies which only depends on mutation. Thus, when comparing different rates of clonality in populations with the same size and realistic mutation rate, the increase of the convergence time to $\overline{F_{IS,\infty }}$ is directly related to *t*_*c*_ until *t*_*c*_ > *t*_*μ*_.

**Table 1:** Convergence times under partial clonality for seven representative example parameter sets

*N* = 100	*c*	*μ*	*t*_*N*_	*t*_*c*_	*t*_*μ*_	*t*_*I*_	*t*_*II*_	*t*_*III*_
**A**	0.0	10^−6^	159	**1**	2.6 × 10^6^	1	1	2.6 × 10^6^
**B**	0.8	10^−6^	159	**25**	2.6 × 10^6^	27	27	2.6 × 10^6^
**C**	0.97	10^−6^	159	**159**	2.6 × 10^6^	174	177	2.6 × 10^6^
**D**	0.99	10^−6^	159	**529**	2.6 × 10^6^	464	498	2.6 × 10^6^
**E**	1.0	10^−6^	159	∞	**2.6 × 10** ^**6**^	38,366	≫ 40,000	2.6 × 10^6^
**F**	1.0	10^−2^	159	∞	**263**	234	138	264
**G**	1.0	10^−1^	159	∞	**25**	25	14	25

Population size *N* = 100 throughout. *Columns: c* – rate of clonality, *F*_*IS*,∞_ = 0 – mutation rate, *t*_*N*_ – genetic drift maximal expected convergence time, *t*_*c*_ – reproduction maximal convergence time, *t*_*μ*_ – mutation maximal convergence time, *t*_*I*_ – convergence time to the mean $\overline{F_{IS,\infty }}$ based on the model in [13], *t*_*II*_ – convergence time to the mean $\overline{F_{IS,\infty }}$ based on our model, *t*_*III*_ – convergence time to full final distribution of $\tilde{{F_{IS}}_{,\infty }}$. *Rows:* example parameter sets (compare Fig. 4). Bold: min (*t*_*c*_, *t*_*μ*_)

Finally, an important observation is also that when approaching the steady state in partially clonal populations, the variations of *F*_*IS*_ increase again as compared to variations expected at the steady states, as the “evolutionary memory” of clonality (sensu [38]) ease the influence of genetic drift on genotype frequencies (Fig. 4). Partial clonal populations that have not yet reached their steady state distribution of $\tilde{{F_{IS}}_{,\infty }}$ may thus show greater deviations from their current exact mean $\overline{F_{IS,t}}$ value than expected for the final $\overline{F_{IS,\infty }}$ values, especially in small population sizes (Additional file 2: Figures S2.8 and S2.9). For example in highly clonal populations (Fig. 4d, e at *t* = 50), the average deviations from the current exact mean $\overline{F_{IS,50}}$ based on ten loci even exceeded ±0.1 the variation yet expected at the steady states.

## Discussion

Our results on the dynamics of *F*_*IS*_ in partially clonal populations add a new dimension – time – to the description of the steady state distribution $\tilde{{F_{IS}}_{,\infty }}$ and its mean $\overline{F_{IS,\infty }}$ derived from previous models [13, 16]. They allow estimating the time needed to reach steady state distributions of genotype frequencies, be there are different or not from the classical Hardy-Weinberg proportions defined for sexual populations. Those estimates not only take into account the hyperbolic relationship between the rate of clonality and the convergence times to HWE [15], but also the effects of two other major evolutionary forces, genetic drift and mutation (Fig. 3).

Thus far, the frequent occurrence of departure from HWE observed in partially clonal species was interpreted as fitting a narrow range of scenario where clonality (heterozygote excesses) and/or selfing (homozygote excesses) would dominate. This study offer a new perspective on these observations, by associating significant *F*_*IS*_ to a much broader range of stable and transient scenario including modest rates of clonality, even in the absence of selfing. These results thus call for increased caution when interpreting these field data alone to make inferences on the importance of clonality or selfing, in the absence of other biological knowledge of the mating system.

Indeed, our work confirms the previous findings of departure from the usual average $\overline{F_{IS,\infty }}=0$ only at very high rates of clonality [13, 14, 17]. However, it also recalls and supports the pioneering work from Marshall and Weir [15] on the speed of convergence towards this $\overline{F_{IS,\infty }}$ in partially clonal populations. We extend the previous results by elucidating how the interplay between rate of clonality, population size (i.e. genetic drift) and mutation affects the nature of the steady state, the time required to reach it and the transient variation of the distribution of *F*_*IS*_ before reaching it. The results reported here show that owing to an increased “evolutionary memory” (sensu [38]) of past genotypic diversity in partially clonal populations, population history such as changes in reproductive mode can produce a transient but possibly long-lasting overrepresentation of *F*_*IS*_ values departing from 0, even under modest rates of clonality. Additionally the variation of *F*_*IS*_, which changes dynamically until the steady state is reached, increases the risk to misestimate the mean $\overline{F_{IS,t,L}}$ unless a high number of loci is used. These findings thus contribute to explaining the frequent reports of negative mean $\overline{F_{IS}}$ in the literature even for species expected to undergo clonal reproduction at intermediate rates. Their otherwise restricted interpretation as a signature of extreme clonality, conceived under the narrow light of the steady state distributions of $\overline{F_{IS,\infty }}$, is thus alleviated. In the following, we further discuss the way this increased “evolutionary memory” and the interaction between rate of clonality, genetic drift and mutation affect the interpretation of $\overline{F_{IS}}$ in natural populations of partially clonal organisms.

### The respective effects of reproduction, mutation and genetic drift in partially clonal populations

Results on the dynamics of genotype frequencies due to each evolutionary process (model parameters) formally demonstrate that:

- Compared to the standard expectation for exclusively sexual populations, clonality only slows down the approach to HWE (Fig. 2b), at a rate depending on other processes such as mutation and genetic drift to which it grants an increased influence.
- mutation, if acting independently at each allelic copy, leads towards *F*_*IS*,∞_ = 0 (Fig. 2c and Additional file 2: Figure S2.3) even for extreme clonal rates, when genetic drift is comparatively negligible (μ > 1/N)
- genetic drift, if not negligible, tends towards genotypic uniformity, i.e. either*. F*_*IS*,∞_ = − 1 (one heterozygous genotype remains) or fixation (one homozygous genotype remains), depending on the initial genotype frequencies (Fig. 2e).

Thus the negative $\overline{F_{IS,\infty }}$ predicted by previous models, and observed in many empirical studies, strongly depend not only on the rate of clonality but also on its interplay with the population size and, to a lesser extent, the mutation rate (Fig. 3). $\overline{F_{IS,\infty }}=0$ is expected at steady state even under high rates of clonality and pure clonality in large populations (Additional file 2, 2.6), as well as in smaller populations when mutation rates are high, as e.g. for microsatellites markers (Fig. 3, “mutation” part of the diagram; Fig. 4f, g). Contrastingly, negative $\overline{F_{IS,\infty }}$ as suggested by [13] are also expected, yet only in smaller clonal populations or when back mutations are neglected (as in [13]), thus only where genetic drift dominates the dynamics of genotype frequencies (Fig. 4d, e; Fig. 3, “genetic drift” part of the diagram).

The maximal convergence time for mutation, *t*_*μ*_, to its steady state situated on the HWE (i.e. $\overline{F_{IS,\infty }}=0$) increases with the number of possible alleles (Fig. 2d). This steady state due to mutation exists regardless of the number of alleles or of the mutation scheme as long as all alleles mutate at constant rates (e.g. [39, 40], rates need not be equal among alleles, compare Additional file 1, 1.3). This result is only violated if mutation depends on the other alleles at the same locus within the same individual, as for gene conversion [41, 42]. In this particular case, which we did not assess here but which promotes homozygote excess, populations should converge with a similar dynamics to *F*_*IS*_ = 1 or to the fixation of one allele.

According to our results, clonal populations dominated by mutation and with no gene conversion (Fig. 3, “mutation” part of the diagram) may distinguish themselves from their counterparts dominated by random mating (Fig. 3, “reproduction” part of the diagram) by the rarity of loci that are fixed for one allele throughout the whole population, rather than by a different mean $\overline{F_{IS}}$. In order to assess the relative importance of clonality, the examination of the distribution of *F*_*IS*_ values among multiple loci and of the proportion of fixed loci would thus be more informative than the mean *F*_*IS*_ value itself.

### The dynamics of *F*_*IS*_ in partially clonal populations: the implications of the long lasting temporal dynamics and of the large interloci variance

Our equations and numerical results demonstrated that the dynamics of genotype frequencies and *F*_*IS*_ are slowed down in partially clonal populations, which therefore retain traces of their past for much longer than their exclusively sexual counterparts. This questions the generic value of the final mean $\overline{F_{IS,\infty }}$ as derived in previous studies [13, 16] as a basis for the interpretation of field data. For at least intermediate rates of clonality, the genetic and genotype composition of the population may indeed reflect population history rather than the present day reproductive system and thus mostly depend on the time since the last disturbance of the population (Fig. 4, Additional file 2, 2.6).

**Fig. 5:**
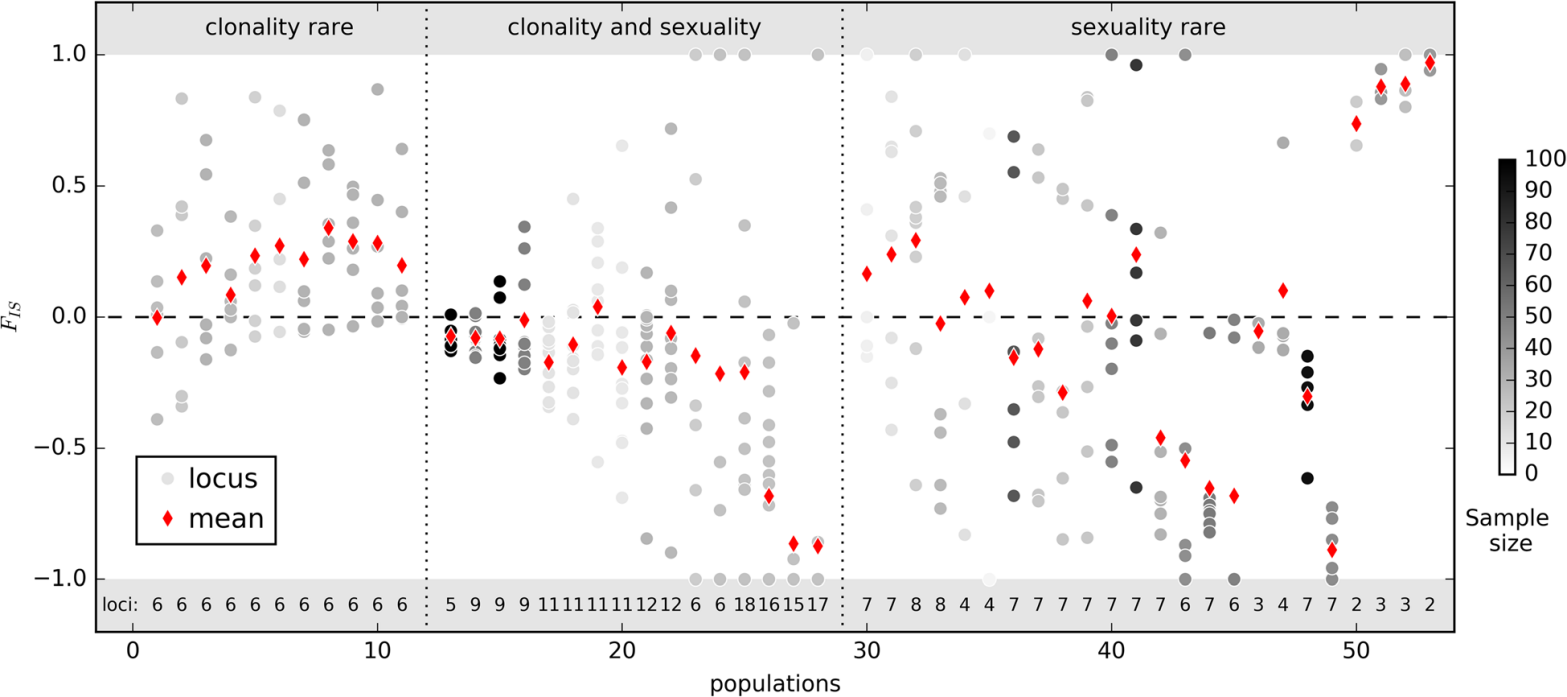
Examples for empirical *F*_*IS*_ values of partially clonal populations compiled from field studies. One population genotyped with SSR per column belonging to 13 species (seven angiosperms, four protists, a sponge and a nematode), based on 13 previous studies (see Additional file 4) selected for their near fit with the assumptions of our model. *F*_*IS*_ values per locus column 32 to 38 and 43 to 48 and 51 were calculated from the reported *H*_*o*_ and *H*_*e*_. Includes populations for which, Populations 14–16 are expected to reproduce using preferential outbreeding during sexual events (self-incompatibility system). Dotted lines separate three groups of populations according to the information given by the authors about their putative rate of clonality, i.e. rarely clonal, frequent clonality and sexuality (including unknown), or rarely sexual. Numbers at the bottom of the plot indicated the number of sampled loci. Number indicated by The hue of each round dot indicates the number of samples (individuals/ramets): light grey: more than 10 ramets genotyped, black: more than 100 ramets genotyped. Red lozenges indicate the mean $\overline{F_{IS,t,L}}$ over all sampled loci per population

The deceleration of the dynamics of *F*_*IS*_ during its approach to the steady state is connected to the hyperbolical increase of *t*_*c*_ (Fig. 2b) and thus much stronger under high rates of clonality. We demonstrated that a comparison of the maximal expected convergence times *t*_*c*_, *t*_*μ*_, *t*_*N*_ can be an efficient means to predict the overall pattern of *F*_*IS*_ dynamics (Figs. 3, 4 and 5, Additional file 2: Figure S2.5). The times *t*_*c*_ and *t*_*μ*_ can even be used to estimate convergence times of the complete model (Table 1): While the time until the steady state distribution of $\tilde{{F_{IS}}_{,\infty }}$ is reached nearly always depends on *t*_*μ*_ in realistic biological conditions, the convergence time to the final mean $\overline{F_{IS,\infty }}$ can be estimated by the minimum of *t*_*c*_ and *t*_*μ*_ (i.e. usually *t*_*c*_ in small populations). If *t*_*N*_ ≪ min(*t*_*c*_, *t*_*μ*_), loci with different initial genotype frequencies may not converge to the same final *F*_*IS*,∞_ value (convergence to genotypic uniformity), so that the expected final $\overline{F_{IS,\infty }}=-1$ cannot be reached within biologically realistic time spans even under nearly pure clonality (Fig. 4e, Additional file 2: Figure S2.5). Though not yet included in our model, perenniality leading to overlapping generations (partial survival of the individuals across generations) is expected to slow down *F*_*IS*_ dynamics even further. If disturbances are sufficiently frequent, e.g. in very instable environments or in populations cyclically changing between exclusive sexual and clonal reproduction [43, 44], the final $\overline{F_{IS,\infty }}$ and $\tilde{{F_{IS}}_{,\infty }}$ based on the currently observed rate of clonality may even never be reached.

Finally, during the convergence toward the final $\tilde{{F_{IS}}_{,\infty }}$, loci may go through intermediate distributions that would be definitely unusual in exclusively sexual populations both in terms of genotype frequencies and *F*_*IS*_ (Fig. 4, Additional file 2: Figure S2.6). Even at modest rates of clonality, the variation of *F*_*IS*_ is increased compared to exclusively sexual populations. Consequently, information from more loci is required to reach an accurate estimate of the mean $\overline{F_{IS,t}}$ in partially clonal populations compared to strictly sexual ones. We found that the variation of *F*_*IS*_ observed during the approach to the steady state distribution may be even greater than predicted based on the final $\tilde{{F_{IS}}_{,\infty }}$, especially when *t*_*N*_ ≫ *t*_*μ*_ (Additional file 2: Figures S2.8 and S2.9).

### Transient *F*_*IS*_ values: a new hypothesis to account for values observed in field data?

We performed a literature review to illustrate the frequent observation of a very wide variety of *F*_*IS*_ values, positive as well as negative, in partially clonal populations (Fig. 5; details in Additional file 4). Field data may be influenced by technical biases, including sampling bias due to an unknown spatial structure of clones, missing rare genotypes due to non-exhaustive sampling, genotyping errors (e.g. undetected null alleles for SSRs) or preferential sampling of loci with near-isoplethy (thus increasing the probability to find negative *F*_*IS*_). Moreover, biological processes other than those in our model may have acted on the data we collected. We therefore applied strict criteria to standardize the dataset we used and retain only those populations that fitted our model best. We present only studies that did include repeated multiloci genotypes in their calculations, published *F*_*IS*_ values (or *H*_*o*_ and *H*_*e*_) per locus and clearly isolated population, and reported data on organisms whose life cycle fits with our model (i.e. dominantly diploid, no cyclic clonality as e.g. in aphids). Only few studies matched these strict criteria [20, 45–56]. We kept studies on species with self-incompatibility systems, as this system of preferential outbreeding has been shown to have very little effect on *F*_*IS*_ at loci physically (nearly) unlinked to the SI genes [57, 58].

Owing to the increased evolutionary memory of past demographic fluctuations, the results of our study open up new possible explanations for the presence, but also the absence, of positive and negative *F*_*IS*_ values (both at individual loci or the mean) in partially clonal populations. On their way back to the steady state distribution, even intermediately clonal populations may transiently exhibit *F*_*IS*,*t*_ values that should be rare in the final steady state distribution $\tilde{{F_{IS}}_{,\infty }}$. Such values can be due to the increased variation of *F*_*IS*_ or echo the past departure from equilibrium due to population history (e.g. demographic bottleneck, change in the rate of clonal reproduction). As an example of how to apply our results, slightly negative mean $\overline{F_{IS,t,L}}$ over loci in some wild cherry populations (populations 14–16 in Fig. 5, [20]), would have suggested almost exclusive clonality when taking the expected mean $\overline{F_{IS,\infty }}$ under equilibrium as the reference. For example, $\overline{F_{IS,t,L}}=-0.083$ the exhaustively sampled population 16 (Fig. 5) with *N* = 247, *μ* ∈ [10^− 3^, 10^− 12^] suggest *c* ≈ 0.98. However, the proportion of repeated multiloci genotypes and inferences from parentage analysis suggested an intermediate rate of clonality instead (*c* ~ 0.5) [20, 59]; based on this value, genotype dynamics would be dominated by random mating (*t*_*c*_ ≈ 10). Using our theoretical results, the observed *F*_*IS*_ distribution and its negative mean could be explained either by extensive logging in the past (population history) or by the fact that only nine loci were analyzed in small populations of about a hundred individuals (increased variation; compare Additional file 2, 2.8 and 2.9).

Our results also suggest ways to further improve the population genetic inferences in natural populations of partial clonals, based on *F*_*IS*_ in connection with other parameters as proposed in [14]. First, other parameters than *F*_*IS*_ may be much more informative when attempting to assess the relative importance of sexual versus clonal reproduction in the dynamics and evolution of partially clonal population. Maximizing the number of loci studied by moving from population genetics to population genomics may help to improve the statistical basis of inferences of population parameters. Second, if pursuing the investigation of the influence of rates of clonality on *F*_*IS*_, rather than focusing exclusively on the mean $\overline{F_{IS,t,L}}$ over loci, the full distribution of $\tilde{{F_{IS}}_{,t,L}}$values per population should be reported and interpreted. Collecting time series of samples may also provide valuable information, as field data normally represent only a “snapshot” of genotype frequencies at a particular point in time, that may or may not be representative of the steady state distribution of the parameters being studied. Using the Markov chain model implemented here, it is not only possible to statistically analyze example trajectories, but also to analytically derive the transition probabilities between two consecutive sets of genotype frequencies for a range of clonal rates, based on population size, mutation rate and number of generations between time series samples. The results of this study open up perspectives for the development of a unified statistical method to infer rates of clonal reproduction, or other population genetic parameters of our model if *c* would be known, based on the analysis of temporal samples. Taking into account the temporal dynamics of genetic descriptors of the populations, including *F*_*IS*_ would therefore help to improve the biological interpretation of values from field data, or to refine methods for estimating the rate of clonality based on a collection of population genetic indices.

## Conclusions

Our results allow reconciling predictions for *F*_*IS*_ under partial clonality from theoretical models, which suggest departures from *F*_*IS*_ = 0 only at nearly pure clonality, with some empirically observed values, which show such departures also where sex is known or suspected to be frequent. Examining the dynamic effects of clonality under the varying influence of mutation and drift showed three main implications for interpreting *F*_*IS*_ in partially clonal populations:

- non-negative *F*_*IS*_, including null values, are not a reliable indicator of the absence of clonal reproduction, as they may occur i) even at steady state under highly frequent but not exclusive clonality, provided the influence of mutation compared to drift in populations is large (*μ* ≫ 1/*N*). Or they may occur ii) transiently under all rates of clonality, a likely situation for many wild populations considering the hyperbolic relationship between the rate of clonality and the time toward convergence reported here.
- negative *F*_*IS*_ values are not a reliable indicator of nearly exclusive clonal reproduction, as significant deviations from *F*_*IS*_ = 0 for multiple loci are also expected after departures from the steady state distribution in partially clonal populations, which generally last longer even if the rate of clonality is only intermediate.
- An increased number of loci are required to maintain the accuracy of DNA-based estimates of population genetic parameters in partially clonal compared to exclusively sexual populations in general, and the study of time series rather than single snapshots of genetic data may lead to more accurate estimates of the rate of clonal reproduction in particular.

## Nomenclature

*F*_*IS*_, inbreeding coefficient represents a correlation coefficient among alleles at a particular locus within polyploid (diploid here) individuals

*t*, current generation (discrete time)

$\overset{\sim }{{\mathrm{F}}_{\mathrm{IS},\mathrm{t}}}$, exact distribution of *F*_*IS*_ at a time *t*

$\overline{{\mathrm{F}}_{\mathrm{IS},\mathrm{t}}}$, mean *F*_*IS*_ at a time t

H_o_, observed heterozygosity

H_e_, expected heterozygosity

F, allele identity within individuals

*Θ*, allele identity within the population

HWE, Hardy-Weinberg equilibrium

N, population size

n, number of alleles at one locus. Alleles can be named as A, a for a biallelic locus or A1, A2, …Ai for locus with more than two alleles

i ≠ j ≠ k ≠ l, indices referring to alleles

*v*_*i*_*,* allele frequency of Ai

g, number of different genotypes at one locus

q_ij_, number of individual of genotype AiAj

*v*_ij_, genotype frequency of genotype AiAj

c, rate of clonal reproduction

s, rate of selfing, set here at 1/N as expected under random mating

μ, mutation rate

α, the probability that an allele does not mutate

β, the probability that an allele mutates into one of the n − 1 others during one generation

X, random variable

P(X), probability of a random variable X

M, transition matrix

ℳ, multinomial distribution

L, numbers of studied polymorphic loci

t_c_, maximal number of generations to convergence due to rate of clonality

t_μ_, maximal number of generations to convergence due to mutation rate

t_N_, maximal number of generations to convergence due to genetic drift

min(t_c_, t_μ_), minimum convergence time between two evolutionary forces

ε, universal “acceptable error” corresponding to one half the minimal change in genotype frequency that would be measurable by exhaustive sampling in a population of finite size N

q_s_, probabilities that two individuals taken at random in the same reproductive subpopulation after migration were sired in the same reproductive subpopulation one generation before

q_d_, probability that two individuals taken at random in different reproductive subpopulations after migration originated from the same subpopulation one generation before

## Additional files

Additional file 1: Mathematical Background. 1.1 Model equations, 1.2 Convergence times – individual parameters, 1.3 Mutation and heterozygosity – multiple alleles, asymmetric mutation rate, 1.4 Genetic drift and heterozygosity – multiple alleles, 1.5 Convergence times – full model, 1.6 General solution and mixing time of Balloux et al. [13] recurrence equations. (DOCX 73 kb) Additional file 2: Figures and Tables. 2.1. Interpretation of *de Finetti* diagrams, 2.2 *De Finetti* landscapes for reproduction, 2.3 *De Finetti* landscapes for mutation, 2.4 *De Finetti* landscapes for genetic drift, 2.5: Dynamics of probability of fixation and distributions of *F*_*IS*_ through time for large population size, low mutation rate 10^−6^ at locus with 10 alleles 2.6: Dynamics of probability of fixation and distributions of *F*_*IS*_ through time for large population size, high mutation rate 10^−3^ at locus with 10 alleles 2.7 Example trajectories over time. 2.8 Sampling error of the mean $\overline{{\mathrm{F}}_{\mathrm{IS},\mathrm{t},\mathrm{L}}}$ according to number of loci (Markov chain). 2.9 Sampling error of the mean $\overline{{\mathrm{F}}_{\mathrm{IS},\mathrm{t},\mathrm{L}}}$ according to number of loci (simulations) Additional tables. 2.1 transition probabilities conditionally to rates of clonality, mutation rates and previous genotypic state. 2.2 Convergence time of genetic drift *t*_*N*_ based on Markov chain absorption time. 2.3: Convergence time of genetic drift *t*_*N*_ based on simulations. 2.4 Effects of different rates of clonality on the dynamics of *F*_*IS*_. (DOCX 10902 kb) Additional file 3: Basic model code in Python. Input *c*, *μ*, *N*, start state and *t* to get the expected probability distribution of model states. (DOCX 30 kb) Additional file 4: Literature review. References and supplementary data for the literature review. “PN” refers to the number of the dataset(s) in Fig. 5, *: *F*_*IS*_ was calculated from *H*_*e*_ and *H*_*o*_. (DOCX 20 kb)
