# Supplementary material for: Rare sex or out of reach equilibrium? The dynamics of FIS in partially clonal organisms
Source: BMC Genet. 2016 Jun 10;17:76. doi: 10.1186/s12863-016-0388-z (PMC4902967; doi:10.1186/s12863-016-0388-z)
Supplement: Additional file 1: — Mathematical Background. 1.1 Model equations, 1.2 Convergence times – individual parameters, 1.3 Mutation and heterozygosity – multiple alleles, asymmetric mutation rate, 1.4 Genetic drift and heterozygosity – multiple alleles, 1.5 Convergence times – full model, 1.6 General solution and mixing time of Balloux et al. [13] recurrence equations. (DOCX 73 kb) [file 12863_2016_388_MOESM1_ESM.docx]

**Additional file 1: Mathematical background**

**A1.1 Model equations**

For compound equations describing the concatenation of at least two processes, substitute the variables for time $t$ in the second equation by the result for time $t+1$ from the first equation.

Symbols and abbreviations

$N$ … population size $c$ … rate of asexual reproduction $\mu$ … mutation rate

$n$ … number of alleles $g$ … number of genotypes $t$ … current generation

$\nu_{i}$ … allele frequency $\nu_{ij}$ … genotype frequency ${q_{ii}=N\nu}_{ii}, {q_{ij}=N\nu}_{ij}$

$i\neq j\neq k\neq l$ … indices referring to alleles $\alpha=1-\mu$ $\beta=\mu/{(n-1)}$

$\mathcal{M}$ … multinomial distribution $X$ … random variable $P$ … probability

$t_{c}, t_{\mu}, t_{N}$ … max. expected number of generations to convergence $\lambda$ … eigenvalue

$\mathcal{S}$ … allele substitution matrix $J$ … matrix of ones $I$ … identity matrix

$H=\sum_{i,j} \nu_{ij}=1-\sum_{i} \nu_{ii}$ $H_{e}=2\sum_{i,j} \nu_{i}\nu_{j}=1-\sum_{i} {\nu_{i}}^{2}$ $\forall$ … “for all” sign

$\varepsilon$ … approximation bias, set to $\varepsilon=1/(2N)$ unless specified otherwise

Mutation

- $n=2$:

$$\left[ \begin{matrix} \nu_{aa} \\ \nu_{aA} \\ \nu_{AA} \end{matrix} \right]_{t+1}=\left[ \begin{matrix} {(1-\mu)}^{2} & (1-\mu)\mu& \mu^{2} \\ 2(1-\mu)\mu& {(1-\mu)}^{2}+\mu^{2} & 2(1-\mu)\mu\\ \mu^{2} & (1-\mu)\mu& {(1-\mu)}^{2} \end{matrix} \right]\left[ \begin{matrix} \nu_{aa} \\ \nu_{aA} \\ \nu_{AA} \end{matrix} \right]_{t}$$

- $n>2$:

$$\nu_{ii, t+1}= \nu_{ii,t}\cdot\alpha^{2}+\sum_{j} \nu_{jj,t}\cdot\beta^{2}+\sum_{j} \nu_{ij,t}\cdot\alpha\beta+\sum_{j,k} \nu_{jk,t}\cdot\beta^{2}$$

$$\nu_{ij,t+1}=\left( \nu_{ii,t}+\nu_{jj,t} \right)\cdot2\alpha\beta+\sum_{k} \nu_{kk,t}\cdot2\beta^{2}+\nu_{ij,t}\cdot\left( \alpha^{2}+\beta^{2} \right)=+\sum_{k,l} \left( \nu_{ik,t}+\nu_{jl,t} \right)\cdot\left( \alpha\beta+\beta^{2} \right)+\sum_{k,l} \nu_{kl,t}\cdot{2\beta}^{2}$$

Reproduction

- $n=2$:

$\left[ \begin{matrix} \nu_{aa} \\ \nu_{aA} \\ \nu_{AA} \end{matrix} \right]_{t+1}=c\left[ \begin{matrix} \nu_{aa} \\ \nu_{aA} \\ \nu_{AA} \end{matrix} \right]_{t}+(1-c)\left[ \begin{matrix} \nu_{a}^{2} \\ 2\nu_{a}\nu_{A} \\ \nu_{A}^{2} \end{matrix} \right]_{t}$, allele frequencies $\left[ \begin{matrix} \nu_{a} \\ \nu_{A} \end{matrix} \right]_{t}=\left[ \begin{matrix} 1 & 0.5 & 0 \\ 0 & 0.5 & 1 \end{matrix} \right]\left[ \begin{matrix} \nu_{aa} \\ \nu_{aA} \\ \nu_{AA} \end{matrix} \right]_{t}$

- $n>2$:

$$\nu_{ii,t+1}={c\nu_{ii,t}+(1-c)\nu}_{i,t}^{2}={c\nu_{ii,t}+(1-c)\cdot\left( \nu_{ii,t}+0.5\sum_{j} \nu_{ij,t} \right)}^{2}$$

$$\nu_{ij,t+1}=c\nu_{ij,t}+2(1-c)\nu_{i,t}\nu_{j,t}=c\nu_{ij,t}+2(1-c)\cdot\left( \nu_{ii,t}+0.5\sum_{k} \nu_{ik,t} \right)\left( \nu_{jj,t}+0.5\sum_{l} \nu_{jl,t} \right)$$

Genetic drift

Note that all $\nu_{ii, t+1}, \nu_{ij, t+1}$ have to fulfill ${{N\nu}_{ii, t+1} \left( =q_{ii, t+1} \right), N\nu}_{ij, t+1}\left( =q_{ij, t+1} \right)\in\mathbb{N}_{0}$.

- $n=2$:

$\left[ \begin{matrix} \nu_{aa} \\ \nu_{aA} \\ \nu_{AA} \end{matrix} \right]_{t+1}=X/N \text{where} X\mathcal{\sim M}\left( N,\left[ \nu_{aa,t},\nu_{aA,t},\nu_{AA,t} \right] \right)$,

*i.e.* for $q_{aa, t+1}, q_{aA, t+1},q_{AA, t+1}\in\mathbb{N}_{0}$ such that $q_{aa, t+1}+q_{aA, t+1}+q_{AA, t+1}=N$:

$$P\left( \left[ \begin{matrix} \nu_{aa} \\ \nu_{aA} \\ \nu_{AA} \end{matrix} \right]_{t+1} | \left[ \begin{matrix} \nu_{aa} \\ \nu_{aA} \\ \nu_{AA} \end{matrix} \right]_{t} \right)=\frac{N!}{\left( q_{aa, t+1} \right)!\cdot\left( q_{aA,t+1} \right)!\cdot\left( q_{AA,t+1} \right)!} \cdot\nu_{aa,t}^{q_{aa, t+1}}\cdot\nu_{aA,t}^{q_{aA,t+1}}\cdot\nu_{AA,t}^{q_{AA,t+1}}$$

- $n>2$:

$\left[ \begin{matrix} \nu_{ii} \\ \nu_{ij} \\ \vdots\end{matrix} \right]_{t+1}=X/N \text{where }X\mathcal{\sim M}\left( N,\left[ \nu_{ii,t},\nu_{ij,t},\ldots\right] \right)$,

*i.e.* for $q_{ii, t+1}, q_{ij, t+1}, \ldots\in\mathbb{N}_{0}$ such that $\sum_{i} q_{ii, t+1}+\sum_{ij} q_{ij, t+1}=N$:

$$P\left( \left[ \begin{matrix} \nu_{ii} \\ \nu_{ij} \\ \vdots\end{matrix} \right]_{t+1} | \left[ \begin{matrix} \nu_{ii} \\ \nu_{ij} \\ \vdots\end{matrix} \right]_{t} \right)=\frac{N!}{\prod_{i} \left( q_{ii, t+1} \right)!\cdot\prod_{i,j} \left( q_{ij, t+1} \right)!} \cdot\prod_{i} \nu_{ii,t}^{q_{ii,t+1}}\cdot\prod_{i,j} \nu_{ij,t}^{q_{ij,t+1}}$$

**A1.2 Convergence times – individual parameters**

Reproduction

As can be easily demonstrated from the reproduction equations in part *A1.1*, neither random mating nor asexual reproduction *per se* change allele frequencies, they only affect the proportion of heterozygous and homozygous genotypes. Let $H_{t}$ denote the observed heterozygosity at time $t$, and $H_{e}$ the expected heterozygosity at $F_{IS}=0$ (convergence domain) for a given set of allele frequencies.

The sum over all equations $\nu_{ij,t+1}=c\nu_{ij,t}+2(1-c)\nu_{i,t}\nu_{j,t}$ can be rewritten as $H_{t+1}=cH_{t}+(1-c)H_{e}$. Inserting this result into the equation for $F_{IS}$ gives:

$$F_{IS,t+1}=\frac{H_{e}-H_{t+1}}{H_{e}}=\frac{H_{e}-(cH_{t}+(1-c)H_{e})}{H_{e}}=c\cdot\frac{H_{e}-H_{t}}{H_{e}}=c\cdot F_{IS,t}$$

This recursive relation can be rewritten as a geometric sequence, $F_{IS,t=x}=c^{x-1}\cdot F_{IS,t=0}$. By defining an “acceptable error” $\varepsilon$, we can also calculate the time $t_{c}$ until it has converged arbitrarily close to $F_{IS}=0$, starting from $\left| F_{IS} \right|=1$, depending on $c$: $\varepsilon=c^{t_{c}-1}$, which transforms to $t_{c}=1+\log_{c} \varepsilon=1+\frac{\log\varepsilon}{\log c}$. For $c=0$, $t_{c}=1$; in contrast, $t_{c}$ is infinite (no convergence) if $c=1$. A good value for $\varepsilon$ may be $1/2N$, half the frequency corresponding to one individual of the population (detection threshold for deviations from $F_{IS}=0)$.

Mutation

For any $n$, mutation between genotypes can be described by a matrix similar to the one given in part *A1.1* or table *A1.3-1*. Thus, the genotype frequency vectors issued from the mutation process converge to the matrix’ dominant eigenvector (eigenvalue 1), and the convergence can be approximated by a geometric sequence whose common ratio corresponds to the matrix’ second largest eigenvalue.

For $n=2$, the eigenvalues of the mutation matrix can be calculated “by hand”: $\lambda=\{1, 1-2\mu, {(1-2\mu)}^{2}\}$, each with a multiplicity of one. The time to convergence, depending on $\mu$, therefore approximately corresponds to $t_{\mu}=1+\log_{(1-2\mu)} \varepsilon=1+\frac{\log\varepsilon}{\log(1-2\mu)}$. Looking at the extreme values of $\mu$,$t_{\mu}=1$ for $\mu=0.5$, and there is no convergence if $\mu=0$.

For $n>2$, the eigenvalues of the mutation matrix can be derived from the eigenvalues of the allele substitution matrix $\mathcal{S}$. If we first consider genotypes as ordered, rather than unordered, pairs of alleles, mutation between them is described by the Kronecker product of $\mathcal{S}$ with itself. The eigenvalues of $\mathcal{S\otimes S}$ are given by the pairwise products $\lambda_{\mathcal{S,}i}\lambda_{\mathcal{S,}j}$ (including $i=j$) of the eigenvalues of $\mathcal{S}$. For the *Jukes-Cantor* substitution model, as $\mathcal{S=}\beta J+\left( \alpha-\beta\right)I$, the eigenvalues of the allele substitution matrix are $\lambda_{\mathcal{S}}=\{\alpha+\left( n-1 \right)\beta, \alpha-\beta\}=\{1, 1-\mu\frac{n}{n-1}\}$ with multiplicities $\{1, n-1\}$. The eigenvalues of the mutation matrix are therefore $\lambda=\{1, 1-\mu\frac{n}{n-1}, {(1-\mu\frac{n}{n-1})}^{2}\}$, and their multiplicities $\left\{ 1, n-1, \frac{n(n-1)}{2} \right\}$ after adjusting for the “unorderedness” of the alleles within genotypes.

Thus, for $n>2$, the second largest eigenvalue reduces to $(1-n\beta)=(1-\mu\cdot n/(n-1))$, and its multiplicity increases to $n-1$. Consequently, the more alleles there are, the longer it takes until mutation has reached its equilibrium.

Genetic drift

If we consider a Markov chain for the states of our model based only on multinomial drawing, all states where only one genotype exists are absorptive. The convergence time $t_{N}$ for genetic drift thus corresponds to the maximum of the expected time until absorption / the expected time until “fixation” of one genotype starting from any non-absorptive (transient) state.

The vector of expected absorption times for a population can be calculated from its fundamental matrix, *i.e.* an identity matrix of corresponding size minus the part of the transition matrix describing only transitions between transient states. For the simplest case of $N=2$ individuals, the fundamental matrix is

$$\left[ \begin{matrix} 0.5 & 0 & 0 \\ 0 & 0.5 & 0 \\ 0 & 0 & 0.5 \end{matrix} \right].$$

The expected absorption times of the three transient states $[0.5, 0.5, 0]$, $[0.5, 0, 0.5]$ and $[0, 0.5, 0.5]$ are given by the column sums of the inverse of this matrix, *i.e.* $(2, 2, 2)$. Consequently, $t_{N}(N=2)=2$, which means that any initially genotypically diverse population would be expected to become uniform after (a maximum of) just two generations.

As $N$ increases, the fundamental matrix is no longer diagonal and the calculation of $t_{N}$ becomes more complex. Also, the vector of expected absorption times is no longer uniform, but depends on each state’s distance to the nearest absorptive state, *e.g.* $[0.9, 0.1, 0.0]$being closer to absorption than $[0.4, 0.3, 0.3]$. In this case, it is easier to obtain such results using simulations consisting in sampling at each generation in previous genotype frequencies to obtain the new generation. For a finite $N$, these distances are divided into quanta of $1/N$ since there can be no fractional individuals; hence the dependence of $t_{N}$ on $N$. Based on the numerical results displayed in Additional Tables 2.2 (from Markov chains) and 2.3 (from simulations), the following linear approximations of this dependence can be made (note that $t_{N}$ not just increases with $N$, but to a lesser extent also with $g$ or $n$):

$$t_{N}\left( n=2; 2\leq N\leq120 \right)=1.6N-1$$

$$t_{N}\left( n=3; 2\leq N\leq15 \right)=1.8N-1.5$$

$t_{N}\left( n=4; 2\leq N\leq8 \right)=1.95N-2$.

For infinite $N$, the underlying multinomial distribution turns into a multinormal, and genetic drift becomes a diffusion process.

**A1.3 Mutation and heterozygosity – multiple alleles, asymmetric mutation rate**

Multiple alleles

*Table A1.3-1:* Mutation rates between diploid genotypes at one locus with four possible alleles A, G, C and T (SNP), based on the Jukes-Cantor substitution model. Mutations from “column” to “row” genotype with $\alpha=(1-\mu)$, $\beta=\mu/3$. Note that all columns sum to one.

|  | Homozygote genotypes | | | | Heterozygote genotypes | | | | | |
| --- | --- | --- | --- | --- | --- | --- | --- | --- | --- | --- |
| $↲$ | AA | GG | CC | TT | AG | AC | AT | GC | GT | CT |
| AA | $\alpha^{2}$ | $\beta^{2}$ | $\beta^{2}$ | $\beta^{2}$ | $\alpha\beta$ | $\alpha\beta$ | $\alpha\beta$ | $\beta^{2}$ | $\beta^{2}$ | $\beta^{2}$ |
| GG | $\beta^{2}$ | $\alpha^{2}$ | $\beta^{2}$ | $\beta^{2}$ | $\alpha\beta$ | $\beta^{2}$ | $\beta^{2}$ | $\alpha\beta$ | $\alpha\beta$ | $\beta^{2}$ |
| CC | $\beta^{2}$ | $\beta^{2}$ | $\alpha^{2}$ | $\beta^{2}$ | $\beta^{2}$ | $\alpha\beta$ | $\beta^{2}$ | $\alpha\beta$ | $\beta^{2}$ | $\alpha\beta$ |
| TT | $\beta^{2}$ | $\beta^{2}$ | $\beta^{2}$ | $\alpha^{2}$ | $\beta^{2}$ | $\beta^{2}$ | $\alpha\beta$ | $\beta^{2}$ | $\alpha\beta$ | $\alpha\beta$ |
| AG | $2\alpha\beta$ | $2\alpha\beta$ | ${2\beta}^{2}$ | ${2\beta}^{2}$ | $\alpha^{2}+\beta^{2}$ | $\alpha\beta+\beta^{2}$ | $\alpha\beta+\beta^{2}$ | $\alpha\beta+\beta^{2}$ | $\alpha\beta+\beta^{2}$ | ${2\beta}^{2}$ |
| AC | $2\alpha\beta$ | ${2\beta}^{2}$ | $2\alpha\beta$ | ${2\beta}^{2}$ | $\alpha\beta+\beta^{2}$ | $\alpha^{2}+\beta^{2}$ | $\alpha\beta+\beta^{2}$ | $\alpha\beta+\beta^{2}$ | ${2\beta}^{2}$ | $\alpha\beta+\beta^{2}$ |
| AT | $2\alpha\beta$ | ${2\beta}^{2}$ | ${2\beta}^{2}$ | $2\alpha\beta$ | $\alpha\beta+\beta^{2}$ | $\alpha\beta+\beta^{2}$ | $\alpha^{2}+\beta^{2}$ | ${2\beta}^{2}$ | $\alpha\beta+\beta^{2}$ | $\alpha\beta+\beta^{2}$ |
| GC | ${2\beta}^{2}$ | $2\alpha\beta$ | $2\alpha\beta$ | ${2\beta}^{2}$ | $\alpha\beta+\beta^{2}$ | $\alpha\beta+\beta^{2}$ | ${2\beta}^{2}$ | $\alpha^{2}+\beta^{2}$ | $\alpha\beta+\beta^{2}$ | $\alpha\beta+\beta^{2}$ |
| GT | ${2\beta}^{2}$ | $2\alpha\beta$ | ${2\beta}^{2}$ | $2\alpha\beta$ | $\alpha\beta+\beta^{2}$ | ${2\beta}^{2}$ | $\alpha\beta+\beta^{2}$ | $\alpha\beta+\beta^{2}$ | $\alpha^{2}+\beta^{2}$ | $\alpha\beta+\beta^{2}$ |
| CT | ${2\beta}^{2}$ | ${2\beta}^{2}$ | $2\alpha\beta$ | $2\alpha\beta$ | ${2\beta}^{2}$ | $\alpha\beta+\beta^{2}$ | $\alpha\beta+\beta^{2}$ | $\alpha\beta+\beta^{2}$ | $\alpha\beta+\beta^{2}$ | $\alpha^{2}+\beta^{2}$ |

The general equations for mutation (part *A1.1*, compare table *A1.3-1* for $n=4$) are:

$$\nu_{ii, t+1}= \nu_{ii,t}\cdot\alpha^{2}+\sum_{j} \nu_{jj,t}\cdot\beta^{2}+\sum_{j} \nu_{ij,t}\cdot\alpha\beta+\sum_{j,k} \nu_{jk,t}\cdot\beta^{2}$$

$$\nu_{ij,t+1}=\left( \nu_{ii,t}+\nu_{jj,t} \right)\cdot2\alpha\beta+\sum_{k} \nu_{kk,t}\cdot2\beta^{2}+\nu_{ij,t}\cdot\left( \alpha^{2}+\beta^{2} \right)=+\sum_{k,l} \left( \nu_{ik,t}+\nu_{jl,t} \right)\cdot\left( \alpha\beta+\beta^{2} \right)+\sum_{k,l} \nu_{kl,t}\cdot{2\beta}^{2}$$

If we sum the frequencies of all homozygous or heterozygous genotypes (*e.g.* sums over upper and lower part of table *A1.3-1*), we get:

$$\left( 1-H \right)_{t+1}=\sum_{i} \nu_{ii,t+1}=\alpha^{2}\sum_{i} \nu_{ii,t}+\left( n-1 \right)\cdot\beta^{2}\sum_{i} \nu_{ii,t}+2\alpha\beta\sum_{i,j} \nu_{ij,t}+\left( n-2 \right)\cdot\beta^{2}\sum_{i,j} \nu_{ij,t}=\left[ \alpha^{2}+\left( n-1 \right)\cdot\beta^{2} \right]\sum_{i} \nu_{ii,t}+\left[ 2\alpha\beta+\left( n-2 \right)\cdot\beta^{2} \right]\sum_{i,j} \nu_{ij,t}$$

$$H_{t+1}=\sum_{i,j} \nu_{ij,t+1}=\left( n-1 \right)\cdot2\alpha\beta\sum_{i} \nu_{ii,t}+\left( \frac{\left( n-1 \right)\left( n-2 \right)}{2} \right)\cdot{2\beta}^{2}\sum_{i} \nu_{ii,t}+\left( \alpha^{2}+\beta^{2} \right)\sum_{i,j} \nu_{ij,t}=+ 2\left( n-2 \right)\cdot\left( \alpha\beta+\beta^{2} \right)\sum_{i,j} \nu_{ij,t}+\left( \frac{\left( n-2 \right)\left( n-3 \right)}{2} \right)\cdot{2\beta}^{2}\sum_{i,j} \nu_{ij,t}=\left[ \left( n-1 \right)\cdot2\alpha\beta+\left( n-1 \right)\left( n-2 \right)\cdot\beta^{2} \right]\sum_{i} \nu_{ii,t}=+\left[ \left( \alpha^{2}+\beta^{2} \right)+2\left( n-2 \right)\cdot\left( \alpha\beta+\beta^{2} \right)+\left( n-2 \right)\left( n-3 \right)\cdot\beta^{2} \right]\sum_{i,j} \nu_{ij,t}$$

As the equilibrium is reached if heterozygosity does not change anymore over time, *i.e.* transitions from homozygous to heterozygous genotypes are as frequent as the inverse, we can drop the time indices and write:

$$\left[ 2\alpha\beta+\left( n-2 \right)\cdot\beta^{2} \right]H=\left[ \left( n-1 \right)\cdot2\alpha\beta+\left( n-1 \right)\left( n-2 \right)\cdot\beta^{2} \right]\left( 1-H \right)$$

After dividing both sides by $\beta$ and re-substituting $\alpha=(1-\mu)$, $\beta=\mu/{(n-1)}$, we get:

$$\left[ 2\left( 1-\mu\right)+\frac{n-2}{n-1}\cdot\mu\right]H=\left[ 2\left( n-1 \right)\cdot\left( 1-\mu\right)+\left( n-2 \right)\cdot\mu\right]\left( 1-H \right)$$

This simplifies to:

$$\left[ 2\left( n-1 \right)-n\mu\right]\left( n-1 \right)^{-1}H=\left[ 2\left( n-1 \right)-n\mu\right]\left( 1-H \right)$$

As $n\geq2$ and $\mu\geq0$, the solution for $H$ is:

$$H=\frac{n-1}{n}$$

This is exactly identical to the expected heterozygosity under $n$-allele HWE for equifrequent alleles. For an infinite number of alleles, $H$ converges to one:

$$\lim_{n\to\infty} H=\lim_{n\to\infty} \frac{n-1}{n}=1.$$

Asymmetric mutation rate

To have “manually” verifiable results, we will again use a two-alleles model for illustration: Let *a* and *A* be two different alleles (DNA nucleotides, SSR copy numbers) with mutation rate $\mu_{a}$ for a 🡪 A and $\mu_{A}$ for A 🡪 a. This corresponds to the following mutation scheme:

| $↲$ | a | A |
| --- | --- | --- |
| a | $1-\mu_{a}$ | $\mu_{A}$ |
| A | $\mu_{a}$ | $1-\mu_{A}$ |

Mutations between the two alleles can then be described by the allele substitution matrix $\mathcal{S}$

$$\mathcal{S=}\left[ \begin{matrix} 1-\mu_{a} & \mu_{A} \\ \mu_{a} & 1-\mu_{A} \end{matrix} \right]$$

which has the dominant eigenvector (final allele frequencies):

$$\left[ \begin{matrix} \nu_{a,\infty} \\ \nu_{A,\infty} \end{matrix} \right]=\left[ \begin{matrix} \frac{\mu_{A}}{\mu_{a}+\mu_{A}} \\ \frac{\mu_{a}}{\mu_{a}+\mu_{A}} \end{matrix} \right]\text{, or} \frac{\nu_{a,\infty}}{\nu_{A,\infty}}=\frac{\mu_{A}}{\mu_{a}}.$$

Assuming that each allele mutates independently, i.e. the mutation rates between genotypes are the product of the mutation rates between alleles, this corresponds to the following mutation scheme at the genotype level:

| $↲$ | aa | aA | AA |
| --- | --- | --- | --- |
| aa | ${(1-\mu_{a})}^{2}$ | $\mu_{A}(1-\mu_{a})$ | ${\mu_{A}}^{2}$ |
| aA | $2\mu_{a}(1-\mu_{a})$ | $\mu_{a}\mu_{A}+(1-\mu_{a})(1-\mu_{A})$ | $2\mu_{A}(1-\mu_{A})$ |
| AA | ${\mu_{a}}^{2}$ | $\mu_{a}(1-\mu_{A})$ | ${(1-\mu_{A})}^{2}$ |

Treating the genotypes as “ordered” (i.e. “*aA”* ≠ “*Aa”*), the mutation rates in the genotype mutation scheme can be directly derived from those in the allele mutation scheme – they correspond to the Kronecker product of $\mathcal{S}$ with itself:

$$\mathcal{K=S\otimes S=}\left[ \begin{matrix} {(1-\mu_{a})}^{2} & \mu_{A}(1-\mu_{a}) & \mu_{A}(1-\mu_{a}) & {\mu_{A}}^{2} \\ \mu_{a}(1-\mu_{a}) & (1-\mu_{a})(1-\mu_{A}) & \mu_{a}\mu_{A} & \mu_{A}(1-\mu_{A}) \\ \mu_{a}(1-\mu_{a}) & \mu_{a}\mu_{A} & (1-\mu_{a})(1-\mu_{A}) & \mu_{A}(1-\mu_{A}) \\ {\mu_{a}}^{2} & \mu_{a}(1-\mu_{A}) & \mu_{a}(1-\mu_{A}) & {(1-\mu_{A})}^{2} \end{matrix} \right]$$

To get from $\mathcal{K}$ to the “true” genotype mutation scheme, rows 2 & 3 that describe mutations resulting in either of the two synonymous heterozygous genotypes have to be summed, and one of the two columns 2 or 3 describing mutations from each of the synonymous genotypes towards all others is then discarded.

The eigenvector of the matrix $\mathcal{K}$ equals the Kronecker product of the eigenvectors of $\mathcal{K}$’s factors, the two identical matrices $\mathcal{S}$:

$\left[ \begin{matrix} \nu_{a,\infty} \\ \nu_{A,\infty} \end{matrix} \right]\otimes\left[ \begin{matrix} \nu_{a,\infty} \\ \nu_{A,\infty} \end{matrix} \right]=\left[ \begin{matrix} \frac{\mu_{A}}{\mu_{a}+\mu_{A}} \\ \frac{\mu_{a}}{\mu_{a}+\mu_{A}} \end{matrix} \right]\otimes\left[ \begin{matrix} \frac{\mu_{A}}{\mu_{a}+\mu_{A}} \\ \frac{\mu_{a}}{\mu_{a}+\mu_{A}} \end{matrix} \right]=\left[ \begin{matrix} \frac{{\mu_{A}}^{2}}{\left( \mu_{a}+\mu_{A} \right)^{2}} & \frac{\mu_{a}\mu_{A}}{\left( \mu_{a}+\mu_{A} \right)^{2}} & \frac{\mu_{a}\mu_{A}}{\left( \mu_{a}+\mu_{A} \right)^{2}} & \frac{{\mu_{a}}^{2}}{\left( \mu_{a}+\mu_{A} \right)^{2}} \end{matrix} \right]^{T}$

Lumping the synonymous heterozygous genotypes together by summing rows 2 & 3 (or columns 2 & 3 of the transposed vector) gives the final genotype frequencies expected under this asymmetric mutation scheme:

$$\left[ \begin{matrix} \nu_{aa,\infty} & \nu_{aA,\infty} & \nu_{AA,\infty} \end{matrix} \right]^{T}=\left[ \begin{matrix} \frac{{\mu_{A}}^{2}}{\left( \mu_{a}+\mu_{A} \right)^{2}} & \frac{2\mu_{a}\mu_{A}}{\left( \mu_{a}+\mu_{A} \right)^{2}} & \frac{{\mu_{a}}^{2}}{\left( \mu_{a}+\mu_{A} \right)^{2}} \end{matrix} \right]^{T}$$

Consequently, the final genotype frequencies will correspond to:

$$\left[ \begin{matrix} \nu_{aa,\infty} & \nu_{aA,\infty} & \nu_{AA,\infty} \end{matrix} \right]^{T}=\left[ \begin{matrix} {\nu_{a,\infty}}^{2} & 2 \nu_{a,\infty}\nu_{A,\infty} & {\nu_{A,\infty}}^{2} \end{matrix} \right]^{T}$$

or HWE for the final allele frequencies. The same procedure can be applied to any arbitrary allelic mutation scheme (numerically for higher numbers of alleles). Thus, independently of the actual mutation rates or the number of possible alleles, mutation schemes that act on each allele independently will lead towards a randomization of the combinations of alleles within individuals, i.e. HWE.

**A1.4 Genetic drift and heterozygosity – multiple alleles**

Whether genetic drift tends to increase or decrease heterozygosity starting from $F_{IS}=0$ depends on the nature (heterozygous or homozygous) of the most frequent genotype. We shall aim to find the range of allele frequencies for which a homozygous genotype is most frequent.

$(I)$ Without loss of generality, we may assume that $\nu_{1}\geq\nu_{2}\geq\nu_{k} \forall k>2$, *i.e.* $\nu_{1}$ and $\nu_{2}$ are the frequencies of the two most frequent alleles (equality included).

$(II)$ As all allele frequencies must sum to one, it follows that $\nu_{1}+\nu_{2}+\sum_{k} \nu_{k}=1$.

$(III)$ As we are only interested in populations at $F_{IS}=0$ (*i.e.* in HWE), $\nu_{11}=\nu_{1}^{2}$ and $\nu_{12}=2\nu_{1}\nu_{2}$.

Because of $(I)$, $\nu_{11}$ will be the frequency of the most frequent homozygote genotype, and $\nu_{12}$ will be the frequency of the most frequent heterozygous genotype in the population. With $(III)$, a homozygous genotype will therefore be the most frequent if and only if $\nu_{1}^{2}>2\nu_{1}\nu_{2}$ in a population with $n\geq2$ alleles at Hardy-Weinberg equilibrium.

Since $\nu_{1}>0$ because of $(I)$, we can divide both sides of the inequality by $\nu_{1}$ and arrive at the condition $\nu_{1}>2\nu_{2}$. Following from $(I)$ and $(II)$, $2\nu_{2}$ is minimal if all allele frequencies except $\nu_{1}$ are equal; thus we can substitute $\nu_{2}=\frac{1-\nu_{1}}{n-1}$ and resolve the inequality to $\nu_{1}>\frac{2}{n+1}$. Thus, for any given $n$, the most frequent genotype at $F_{IS}=0$ will be homozygous if the frequency of the most frequent allele is greater than twice the frequency of the second-most frequent allele, and at least greater than $\frac{2}{n+1}$. As $n$ decreases whenever one allele is lost by genetic drift, this minimal frequency increases and the range where a homozygous genotype is favored decreases.

**A1.5 Convergence times – full model**

Convergence time to the mean $\bar{F_{IS,\infty}}$**:**

Using the basic equation of our Markov chain model

$$\vec{x}_{t}=M^{t}\vec{x}_{0}$$

where $\vec{x}_{0}$ is the start state vector, $\vec{x}_{t}$ the vector of state probabilities and $M$ the transition matrix (based on $N, \mu, c$), we iteratively calculated the difference between the mean $\bar{F_{IS,t}}$ for the two start states $F_{IS,0}=1, \nu_{a}=\nu_{A}$ and $F_{IS,0}=-1$ at each time step. We considered the mean $\bar{F_{IS,\infty}}$ converged when this difference passed below $\varepsilon=1/(2N)$.

Convergence time to full final distribution of $\tilde{F_{IS,\infty}}$*:*

Similar to our derivation of $t_{\mu}$, the convergence time of the full model can be approximated using the transition matrices’ second largest eigenvalue (Markov chain mixing time approach), $\lambda_{2}$, which we derived numerically. The time to convergence is then $t_{III}=1+\log_{\lambda_{2}} \varepsilon$. Interestingly, we found that $\lambda_{2}=(1-2\mu)$ in all cases we tested. This appears to be a parallel to the model presented in Balloux et al. 2003 (see below – as explained in part *A1.2*, our value $\lambda_{2}=(1-2\mu)$ is a special case of $(1-\mu\frac{n}{n-1})$ which converges to ($1-\mu)$ for $n\to\infty$as in the model from Balloux et al. 2003).

Model from Balloux et al. 2003

Convergence time to the mean $\bar{F_{IS,\infty}}$*:*

Using the model equation (from Balloux et al. 2003, equation 5 & 6):

$$\left[ \begin{matrix} F_{t+1} \\ \Theta_{t+1} \end{matrix} \right]=\left( 1-\mu\right)^{2}\left( \left[ \begin{matrix} c+\frac{1-c}{2N} & (1-c)\left( 1-\frac{1}{N} \right) \\ \frac{1}{2N} & 1-\frac{1}{N} \end{matrix} \right]\left[ \begin{matrix} F_{t} \\ \Theta_{t} \end{matrix} \right]+\left[ \begin{matrix} \frac{1-c}{N} \\ \frac{1}{2N} \end{matrix} \right] \right)$$

where $F_{t}$ represents the observed and $\Theta_{t}$ the expected homozygosity at time $t$, we iteratively calculated the difference between the mean $\bar{F_{IS,t}}$ for the two start states ${[F,\Theta]}_{0}=\left[ 1, 0.5 \right](F_{IS,0}=1, \nu_{a}=\nu_{A})$ and ${[F,\Theta]}_{0}=\left[ 0, 0.5 \right](F_{IS,0}=-1)$ at each time step. We considered the mean $\bar{F_{IS,\infty}}$ converged when this difference passed below $\varepsilon=1/(2N)$.

**A1.6 Extending Balloux et al. (2003) equations to get their general solution considering one finite-sized population and its mixing time**

From Balloux et al. (2003), equation 6 p.1637 derived from equation 2 in Rousset (1996) define the recurrence equations to numerically describe how allelic identities within population ($F$) and between alleles from two different individuals ($\theta$) evolve from one generation to the next:

$Q_{t+1}=\gamma G.Q_{t}+\gamma D$ eq. 1

where $\gamma=1-u^{2}$ with $u$ is the mutation rate for all alleles, $G$ the transition matrix holding the probalistic changes of allelic identities over one generation, $D$ the constant column vector and $Q_{t}$ a column vector holding the probabilities of allelic identities at generation $t$.

From equation 5 p.1636 (Balloux et al. 2003), considering only one finite population (of size *N*), we obtain:

$Q_{t+1}=\left[ \begin{matrix} F_{t+1} \\ \theta_{t+1} \end{matrix} \right]=\gamma\left[ \begin{matrix} c+\frac{1-c}{2N} & (1-c)(1-\frac{1}{N}) \\ \frac{1}{2N} & 1-\frac{1}{N} \end{matrix} \right].\left[ \begin{matrix} F_{t} \\ \theta_{t} \end{matrix} \right]+\gamma\left[ \begin{matrix} \frac{1-c}{2N} \\ \frac{1}{2N} \end{matrix} \right]$ eq. 2

As propose in equation 7 p. 1637 (Balloux et al. 2003), our equation 2 converge after an infinite number of time step (cited as “*equilibrium*”, equation 7, 1637 in Balloux et al. 2003) to $Q^{*}=\left[ \begin{aligned} F^{*} \\ \theta^{*} \end{aligned} \right]={\gamma.\left( I-\gamma G \right)}^{-1}.D$ where $I$ is the identity matrix, here of square dimension.

In one finite population, the steady state of this equation is:

$Q^{*}=\left[ \begin{aligned} F^{*} \\ \theta^{*} \end{aligned} \right]=\frac{\gamma}{p1}\left[ \begin{matrix} 1-\gamma.\left( 1-\frac{1}{N} \right) & \gamma.\left( 1-c \right).\left( 1-\frac{1}{N} \right) \\ \frac{\gamma}{2N} & 1-\gamma\left( c+\frac{1-c}{2N} \right) \end{matrix} \right].\left[ \begin{matrix} \frac{1-c}{2N} \\ \frac{1}{2N} \end{matrix} \right]$ eq.3

where $p1$ stands for the determinant of $I-\gamma G$ and is equal to $p1=\left[ 1-\gamma.\left( c+\frac{1-c}{N} \right) \right].\left[ 1-\gamma.\left( 1-\frac{1}{N} \right) \right]-\frac{\gamma^{2}}{2N}.\left( 1-c \right).\left( 1-\frac{1}{N} \right)$

From those recurrence equations (eq.2) and their steady state defined (eq.3), we follow the classical method of diagonalization (see ^1^) to formalize the general solution of this autonomous system of homogeneous difference equations.

We thus wrote the analytical equations of $\left[ \begin{matrix} F_{t} \\ \theta_{t} \end{matrix} \right]$, the allelic identities within individuals and population at time *t*, as a function of $\left[ \begin{matrix} F_{0} \\ \theta_{0} \end{matrix} \right]$, the allelic identities found at a starting time $t=0$:

$\left[ \begin{aligned} F_{t} \\ \theta_{t} \end{aligned} \right]=\left[ \begin{aligned} F^{*} \\ \theta^{*} \end{aligned} \right]+\left[ \begin{matrix} \lambda_{2}^{t}.i_{1}-\lambda_{1}^{t}.i_{2} & \frac{i_{1}.i_{3}}{2\gamma}\left( \lambda_{1}^{t}-\lambda_{2}^{t} \right) \\ \frac{1}{r}\left( \lambda_{2}^{t}-\lambda_{1}^{t} \right) & \left. \lambda_{1}^{t}.i_{1}-\lambda_{2}^{t}.i_{2} \right. \end{matrix} \right].\left[ \begin{aligned} F_{0}-F^{*} \\ \theta_{0}-\theta^{*} \end{aligned} \right]$ eq. 4

where$r$, $i_{1}$, $i_{2}$ and $i_{1}$ are coefficient that only depend on the rate of clonality, the mutation rate and the population size. They are defined as

$r=\sqrt{\left( 1+c \right)^{2}+4N.{\left( N-1 \right).\left( 1-c \right)}^{2}}$,

$\begin{matrix} i_{1}=\frac{2+\left( 1-c \right).\left( 1-2N \right)}{2.r}+\frac{1}{2} \\ i_{2}=\frac{2+\left( 1-c \right).\left( 1-2N \right)}{2.r}-\frac{1}{2} \\ i_{3}=\gamma. \left[ 2+\left( 1-c \right).\left( 1-2N \right) \right]-\gamma.r \end{matrix}$ and

where $\lambda_{1}$ and $\lambda_{2}$ are the two eigenvalues of the $\gamma G$ matrix:

$\begin{matrix} \lambda_{1}=\frac{\gamma.\left( 2N-1 \right).\left( 1+c \right)-\gamma\sqrt{\left( 1-2N \right)^{2}.\left( 1+c \right)^{2}-16.c.N.(N-1)}}{4N} \\ \lambda_{2}=\frac{\gamma.\left( 2N-1 \right).\left( 1+c \right)+\gamma\sqrt{\left( 1-2N \right)^{2}.\left( 1+c \right)^{2}-16.c.N.(N-1)}}{4N} \end{matrix}$.

From eq. 4, no direct calculation of mixing time can be formalized as the process goes asymptotically to its steady state. We thus defined an acceptable error *ε,* typically $1/N$, so that the mixing time is the time at which $\left[ \begin{matrix} \lambda_{2}^{t}.i_{1}-\lambda_{1}^{t}.i_{2} & \frac{i_{1}.i_{3}}{2\gamma}\left( \lambda_{1}^{t}-\lambda_{2}^{t} \right) \\ \frac{1}{r}\left( \lambda_{2}^{t}-\lambda_{1}^{t} \right) & \left. \lambda_{1}^{t}.i_{1}-\lambda_{2}^{t}.i_{2} \right. \end{matrix} \right].\left[ \begin{aligned} F_{0}-F^{*} \\ \theta_{0}-\theta^{*} \end{aligned} \right]\leq\varepsilon$. It can numerically be computed given a population size *N*, a rate of clonality *c*, a mutation rate *u* and a starting state of the system $\left[ \begin{matrix} F_{0} \\ \theta_{0} \end{matrix} \right]$ from which we want to compute the mixing time to go to the steady state $\left[ \begin{aligned} F^{*} \\ \theta^{*} \end{aligned} \right]$.

References

Balloux François, Lehmann Laurent, de Meeûs Thierry (2003). The population genetics of clonal and partially clonal diploids. *Genetics*, **164**:1635-1644.

Rousset François (1996). Equilibrium values of measures of population subdivision for stepwise mutation processes. *Genetics*, **142**:1357-1362.

^1^Sub-Appendix: Diagonalization and mixing time:

From the system of recurrence equations $Q_{n}=A.Q_{n-1}+b$, we want an analytical formula that is a function of $Q_{0}$ the initial condition of the system and $Q^{*}$ the steady state of the system. We can write $Q_{n}=A.Q_{n-1}+b$ as $Q_{n}-Q^{*}=A.\left( Q_{t}-Q^{*} \right)$ since $Q^{*}=\left( I+A \right)^{-1}.b$ where $I$ is the identity matrix of appropriate order. By recurrence, $Q_{n}-Q^{*}=A.\left( Q_{n-1}-Q^{*} \right)=A^{n}.\left( Q_{0}-Q^{*} \right)$ which results into $Q_{n}=Q^{*}+A^{n}.\left( Q_{0}-Q^{*} \right)$. Thus the perturbation $A^{n}.\left( Q_{0}-Q^{*} \right)$ exponentially goes to zero at a speed that only depends on its highest absolute eigenvalue.
