# Supplementary material for: Rare sex or out of reach equilibrium? The dynamics of FIS in partially clonal organisms
Source: BMC Genet. 2016 Jun 10;17:76. doi: 10.1186/s12863-016-0388-z (PMC4902967; doi:10.1186/s12863-016-0388-z)
Supplement: Additional file 2: — Figures and Tables. 2.1. Interpretation of de Finetti diagrams, 2.2 De Finetti landscapes for reproduction, 2.3 De Finetti landscapes for mutation, 2.4 De Finetti landscapes for genetic drift, 2.5: Dynamics of probability of fixation and distributions of F IS through time for large population size, low mutation rate 10−6 at locus with 10 alleles 2.6: Dynamics of probability of fixation and distributions of F IS through time for large population size, high mutation rate 10−3 at locus with 10 alleles 2.7 Example trajectories over time. 2.8 Sampling error of the mean $\overline{{\mathrm{F}}_{\mathrm{IS},\mathrm{t},\mathrm{L}}}$FIS,t,L¯ according to number of loci (Markov chain). 2.9 Sampling error of the mean $\overline{{\mathrm{F}}_{\mathrm{IS},\mathrm{t},\mathrm{L}}}$FIS,t,L¯ according to number of loci (simulations) Additional tables. 2.1 transition probabilities conditionally to rates of clonality, mutation rates and previous genotypic state. 2.2 Convergence time of genetic drift t N based on Markov chain absorption time. 2.3: Convergence time of genetic drift t N based on simulations. 2.4 Effects of different rates of clonality on the dynamics of F IS. (DOCX 10902 kb) [file 12863_2016_388_MOESM2_ESM.docx]

**Additional file 2: Figures and Tables**

**

**Figure 2.1:** Scheme showing how to read *de Finetti* diagrams.

*De Finetti* diagrams are ternary plots that provide a compact and non-redundant representation of genotype (perpendicular distances from sides), allele (horizontal coordinate) and homo-heterozygote (vertical coordinate) frequencies for a single locus with two alleles. All combinations of genotype counts ($\left( q_{aa}, q_{aA}, q_{AA} \right)$, states of our model) correspond to discrete points on the *de Finetti* diagram, with the distance between neighboring states equal to $1/N$. All states for which $F_{IS}=0$ are on a parabola passing through the fixation states (baseline corners of the triangle) that culminates at the genotype frequencies [0.25, 0.5, 0.25] (vertical height midpoint of the triangle). Points “above” the parabola have negative, “below” the parabola positive $F_{IS}$ values. For each point, the diagram thus allows to simultaneously track e.g. the observed heterozygosity (perpendicular distance from baseline to point), the expected heterozygosity (perpendicular distance from baseline to the Hardy-Weinberg parabola for the given allele frequencies) and the maximum possible heterozygosity (perpendicular distance from baseline to the “upper” side of the triangle for the given allele frequencies), as well as fixation of an allele and the current number of homo-zygote/heterozygote genotypes (central part / sides / corners of the diagram).

Due to the discreteness of individuals, the exact Hardy-Weinberg genotype frequencies cannot be reached for many combinations of allele frequencies. Instead, the states closest to HWE possess slight homozygote or heterozygote excess. Near fixation, the expected heterozygosity and the maximum possible heterozygosity converge, so that excess heterozygosity is no longer distinguishable from HWE. This situation first occurs when the difference between maximum possible and expected heterozygosity passes below $1/N$ (frequency equivalent of one individual). The maximum possible heterozygosity equals $\max\left( \nu_{aA} \right)=2\left( 1-\nu_{a} \right)$ if *a* is the most frequent allele, and the expected heterozygosity equals $\exp\left( \nu_{aA} \right)=H_{e}=2\nu_{a}\left( 1-\nu_{a} \right)$, so that $\frac{1}{N}=2{(1-\nu_{a})}^{2}$ and finally $\nu_{a}=1-\sqrt{1/2N}$ for the frequency of the most frequent allele. If any one allele exceeds this frequency, it is considered nearly fixed. A similar situation where expected and maximal heterozygosity become indistinguishable occurs when the number of different alleles goes towards $2N$, its maximum in a finite population: if there are more than $N$ nearly equally frequent alleles, the difference between maximum possible and expected heterozygosity passes below $1/N$, i.e. graphically the vertex of the (multi-dimensional equivalent of the) Hardy-Weinberg parabola nearly “touches” the states where all individuals of the population are heterozygous.

To visualize the expected changes in the three genotype frequencies (ternary plot coordinates) through time, starting from any possible combination of genotype counts (point/state), we constructed “*De Finetti* landscapes”, i.e. three-dimensional plots where the “height” of each point in the landscape is proportional to the sum of squared genotype frequency changes expected per time step when starting from the respective state. As in classical mechanics, the height of each point in the landscape is thus proportional to the square of the speed with which it is left. This is the basis for an analogy with the natural world that makes these plots intuitively interpretable: one can imagine a population as a small ball “rolling” from “hilltops” towards “valleys”, changing its genotype frequencies according to this displacement within the ternary plot. The point(s) with zero height thus correspond(s) to the final expected state(s) for the respective parameter combinations (reproductive mode, mutation and genetic drift). The flatter the landscape, the longer it takes to reach these states.

*
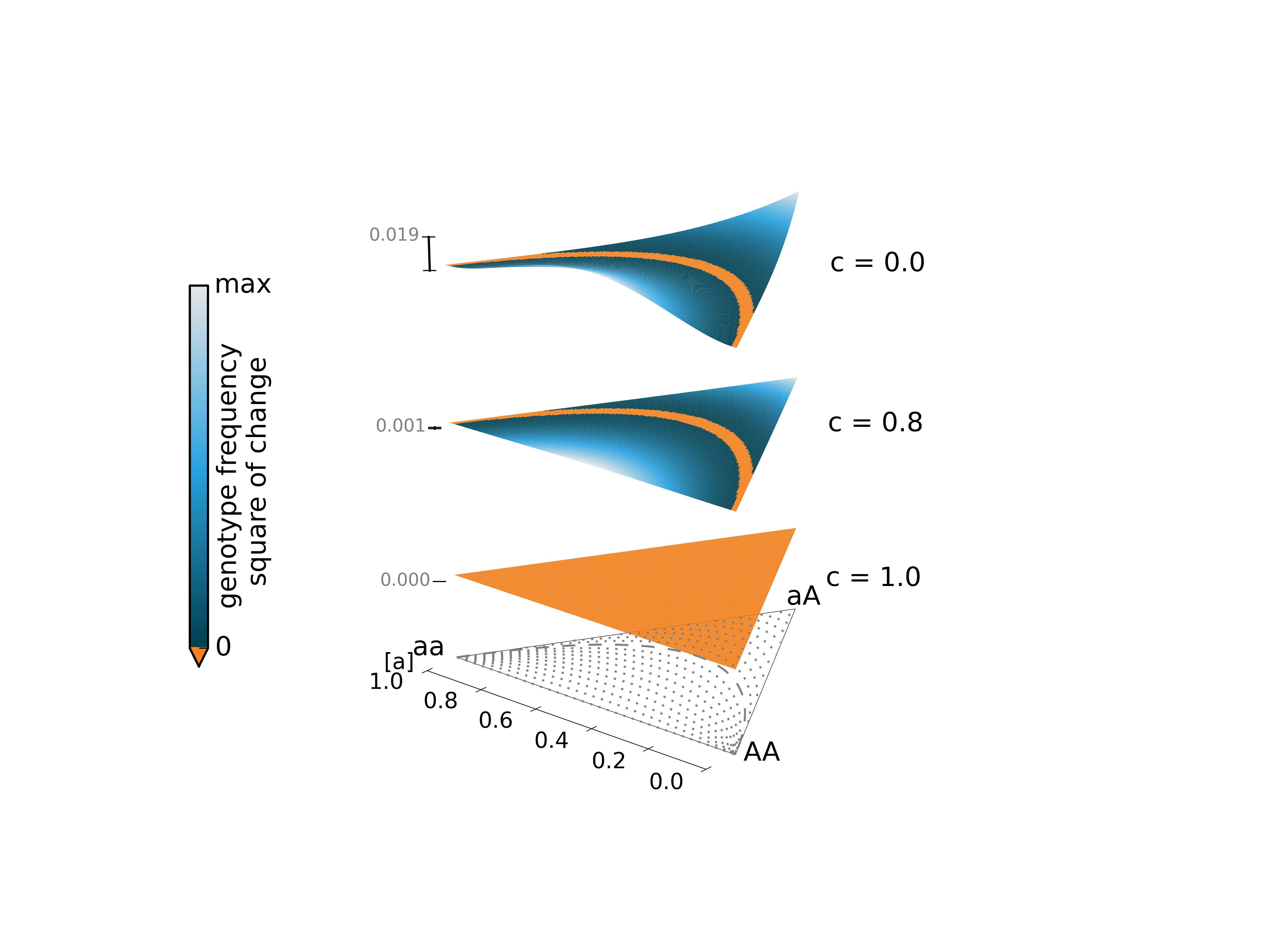
*

**Figure 2.2:** *De Finetti* landscapes for reproduction*.* Increasing the rate of clonality $c$ flattens the landscape (increased time to final expected states), but does not change the final expected states (orange parabola: HWE, $F_{IS}=0$) except if the population is completely clonal $c=1.0$.

*
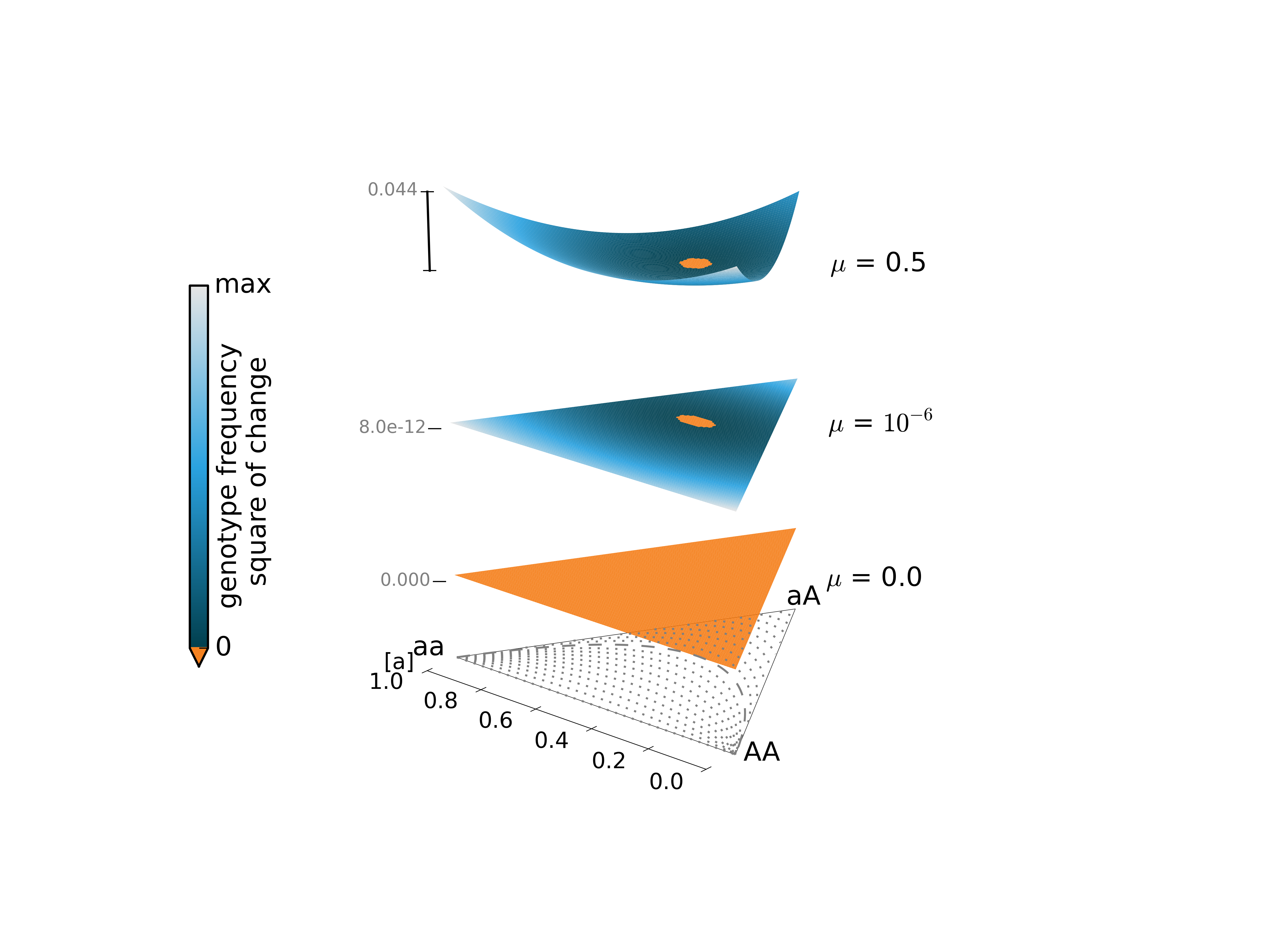
*

**Figure 2.3:** *De Finetti* landscapes for mutation. Decreasing the mutation rate $\mu$ flattens the landscape (increased time to final expected states), but does not change the final expected states (orange dot: HWE, $F_{IS}=0$ for equal allele frequencies, $\nu_{a}=\nu_{A}=0.5$) except if there is no mutation, $\mu=0$.

*
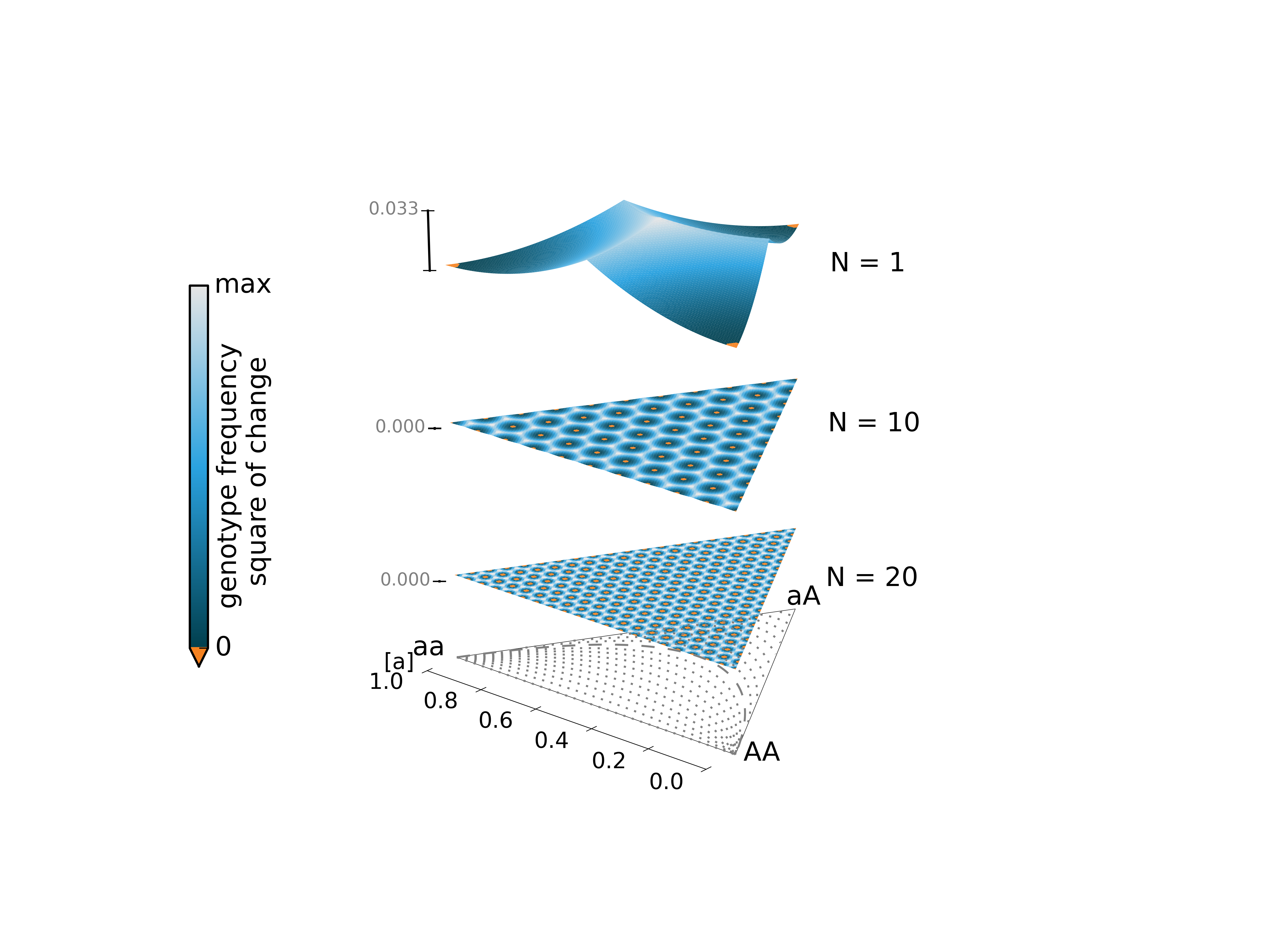
*

*
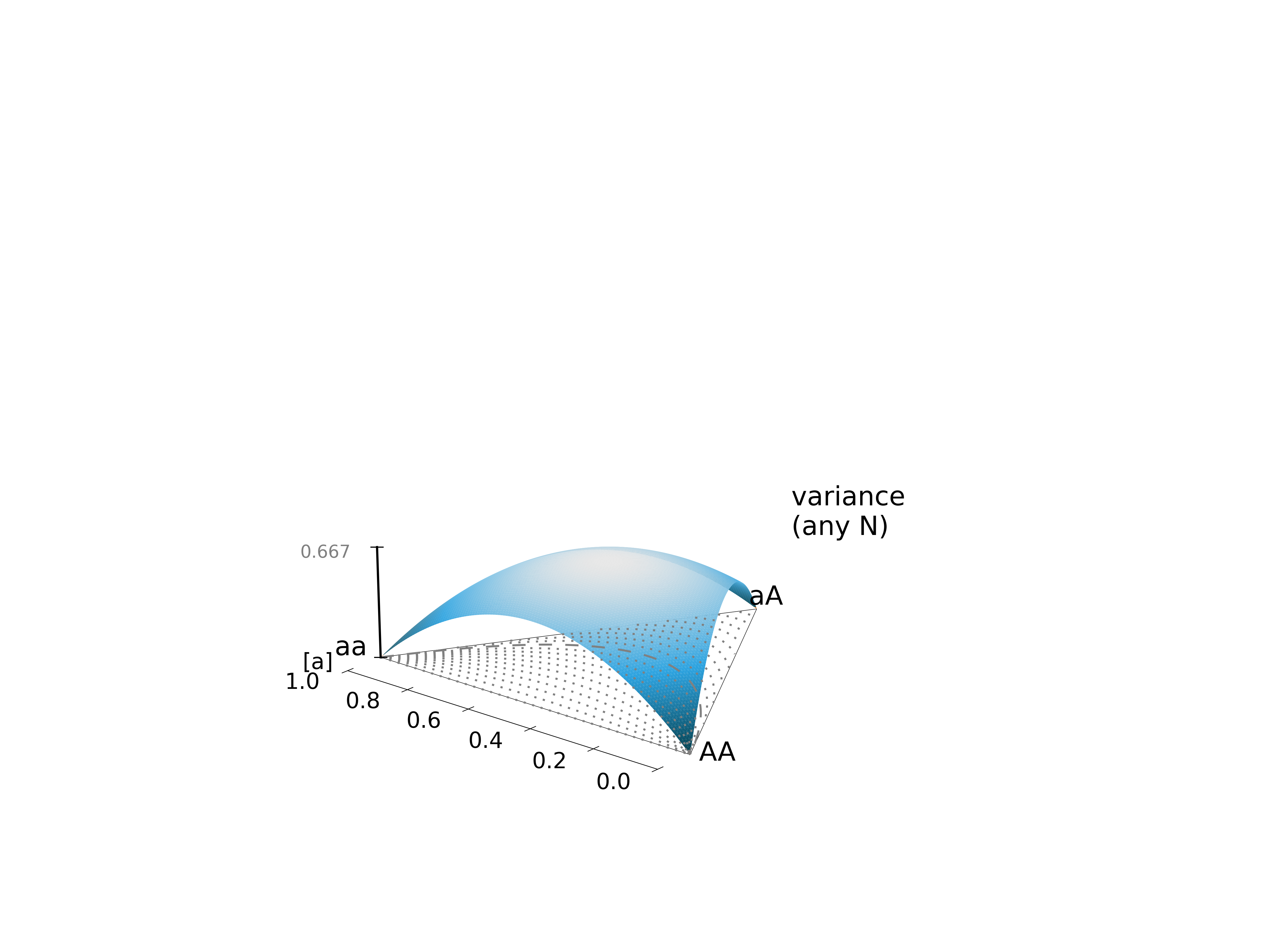
*

**Figure 2.4:** *De Finetti* landscapes for genetic drift. Increasing the population size $N$ increases the density of points with zero expected change in the diagram – for any state, the population is most likely to remain where it was (same genotype frequencies) in the next time step. However, genotype dynamics due to genetic drift can be explained by the variance $Var(X)= \sum_{i} \nu_{ii}(1-\nu_{ii})+ \sum_{i,j} \nu_{ij}(1-\nu_{ij})$around this expectation, which is highest in the center (genotype frequencies [1/3, 1/3, 1/3]) and zero at the corners of the triangle. This means that the direction of random genotype frequency changes due to genetic drift is least predictable if all genotypes are equally frequent, and all random change will cease if the frequency of one genotype becomes one (“fixation” of a genotype). Note that the corresponding co-variances are usually non-zero, as the genotype frequencies are interdependent.

**
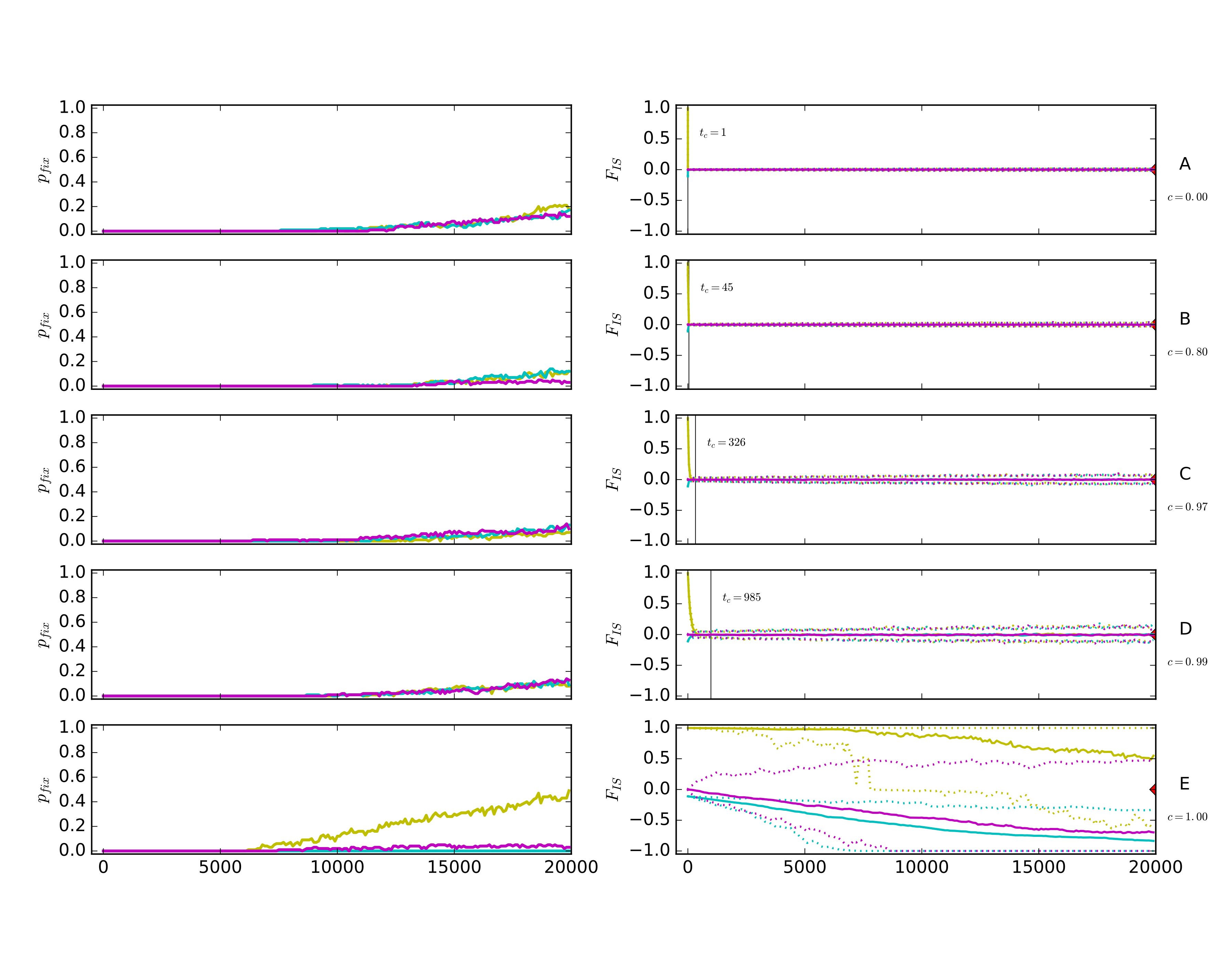
**

**Figure 2.5:** Dynamics of probability of fixation $p_{fix}$ and $F_{IS}$ through time for five representative example parameter sets. Single loci with ten alleles. Colors represent different start states (yellow: $F_{IS,0}=1$ for isoplethic alleles, magenta: $F_{IS,0}=0$ for isoplethic alleles, cyan: all heterozygotes with isoplethic alleles, $F_{IS,0}=-0.1$), with their respective mean (continuous line) and 95% confidence interval (dotted lines) based on 100 repetitions. Vertical lines represent $t_{c}$ (continuous); $t_{\mu}>8.9*{10}^{6}$ and $9900<t_{N}<19800$. Red triangles at $t=200$ indicate the mean $\bar{F_{IS,\infty}}$ according to [13]. Model parameters – $\mu={10}^{-6}$, $N=9900,$ A: $c=0$, B:$c=0.8$, C: $c=0.97$, D: $c=0.99$, E: $c=1.0$. Only data for generation 0, 1, 45, 100 and each following 100^th^ generation are plotted.


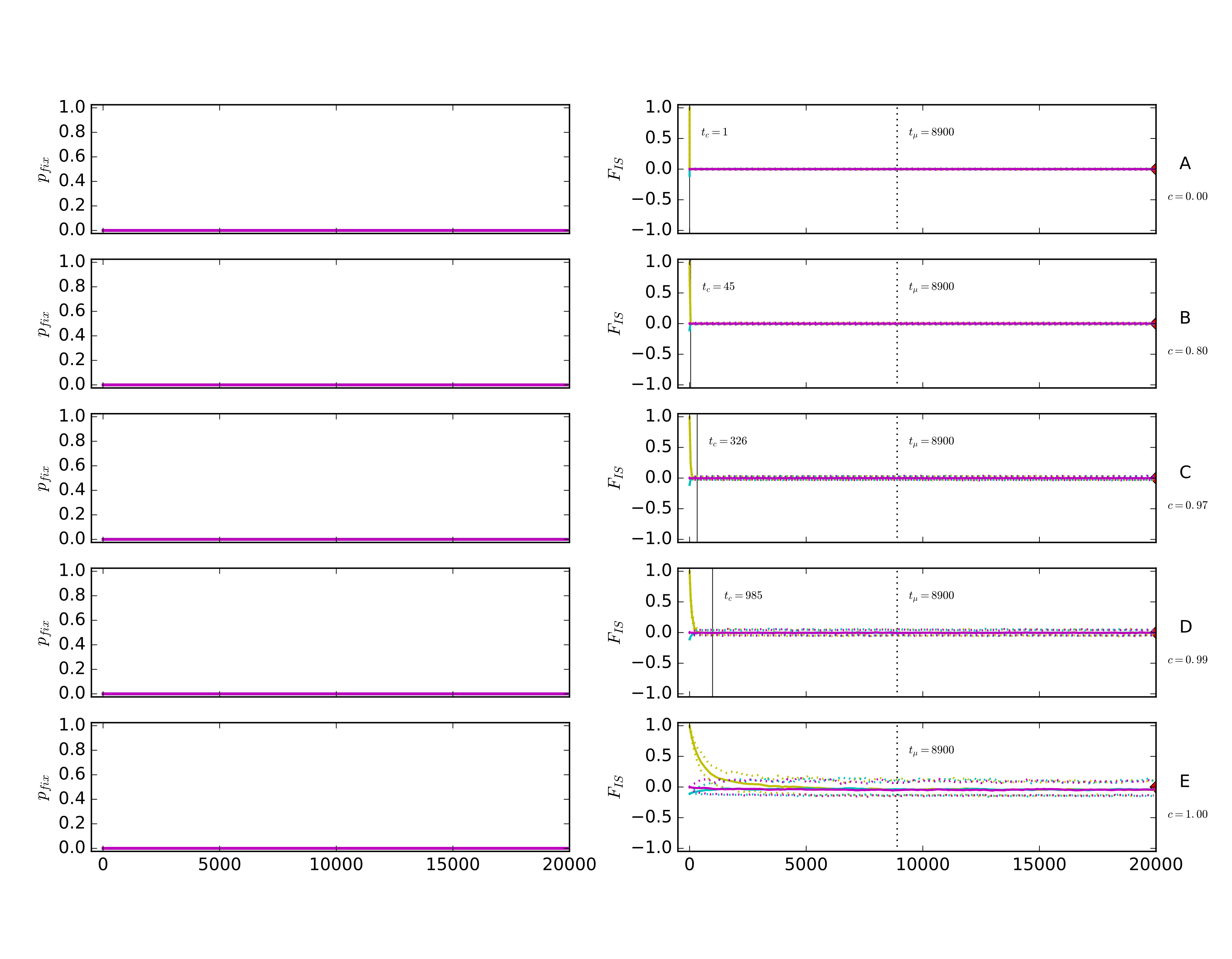


**Figure 2.6:** Dynamics of probability of fixation $p_{fix}$ and $F_{IS}$ through time for five representative example parameter sets. Single loci with ten alleles. Colors represent different start states (yellow: $F_{IS,0}=1$ for isoplethic alleles, magenta: $F_{IS,0}=0$ for isoplethic alleles, cyan: all heterozygotes with isoplethic alleles, $F_{IS,0}=-0.1$), with their respective mean (continuous line) and 95% confidence interval (dotted lines) based on 100 repetitions. Vertical lines represent $t_{c}$ (continuous) and $t_{\mu}$ (dotted); $9900<t_{N}<19800$. Red triangles at $t=200$ indicate the mean $\bar{F_{IS,\infty}}$ according to [13]. Model parameters – $\mu={10}^{-3}$, $N=9900,$ A: $c=0$, B:$c=0.8$, C: $c=0.97$, D: $c=0.99$, E: $c=1.0$. Only data for generation 0, 1, 45, 100 and each following 100^th^ generation are plotted.

*
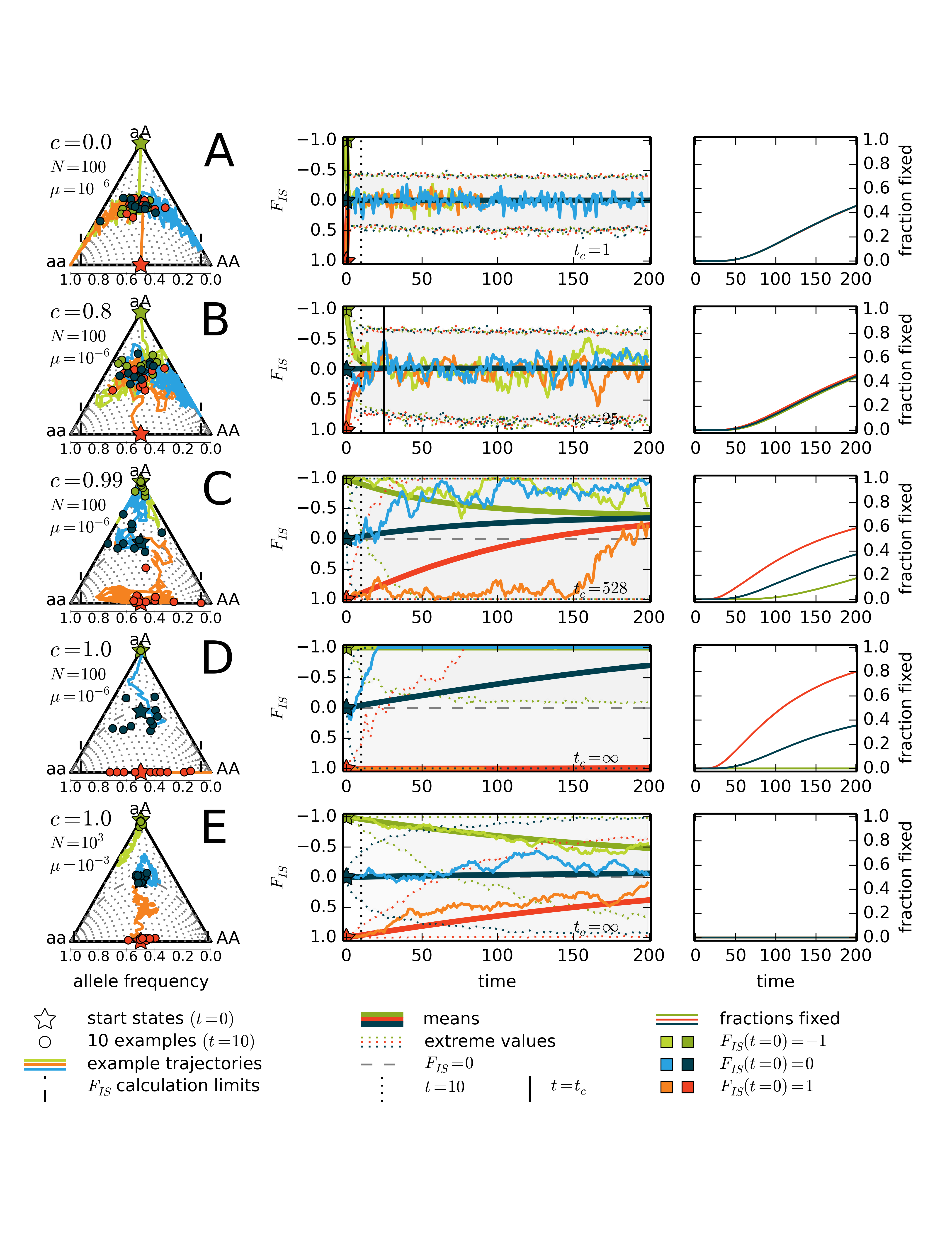
*

**Figure 2.7:** Example trajectories over time for different parameter sets $(c, \mu, N)$. Single loci with two alleles. Overview of interpretation see table 2.2. Color codes – start states: In *light/dark green* (lines/dots/stars), trajectories that started at $F_{IS,0}=-1$ (all individuals heterozygotes i.e both allele frequencies 0.5); in *light/dark blue* (lines/dots/stars), trajectories that started at $F_{IS,0}=0$ and $\nu_{a,0}=\nu_{A,0}=0.5$ (Hardy-Weinberg proportions, both allele frequencies 0.5); and in *red and orange* (lines/dots/stars), trajectories that started at $F_{IS,0}=1$ and $\nu_{a,0}=\nu_{A,0}=0.5$ (all individuals homozygotes, both allele frequencies 0.5). Rows – parameter sets: A, exclusive sexuality, $c=0.0$; B and C, partial clonality with $c=0.8$ and $c=0.99$ respectively; D, exclusive clonality, $c=1.0$*,* low mutation rate and small population; E, exclusive clonality, $c=1.0$*,* high mutation rate and big population. Columns – diagnostic plots: *Left:* *De Finetti* diagrams showing one example trajectory (line) traced over 200 generations and ten example states at $t=10$ (dots) per start state (colors/stars: start states). $F_{IS}$ were not calculated for states outside the vertical dashed black lines (near-fixation, frequency of one allele exceeds $1-\sqrt{1/(2N)}$). *Central:* corresponding dynamics of $F_{IS}$ over 200 generations illustrated by one example trajectory (thin/light line), the mean over 10^5^ trajectories (heavy/dark line) and the range (shaded area delimited by dotted lines) for the three start states (stars); horizontal dashed grey line indicates $F_{IS}=0$; vertical black lines correspond to $t=10$ (dotted) and $t_{c}$ (solid). *Right:* corresponding fraction of trajectories at fixation for one allele, out of 10^5^ trajectories over 200 generations for the three start states.

**
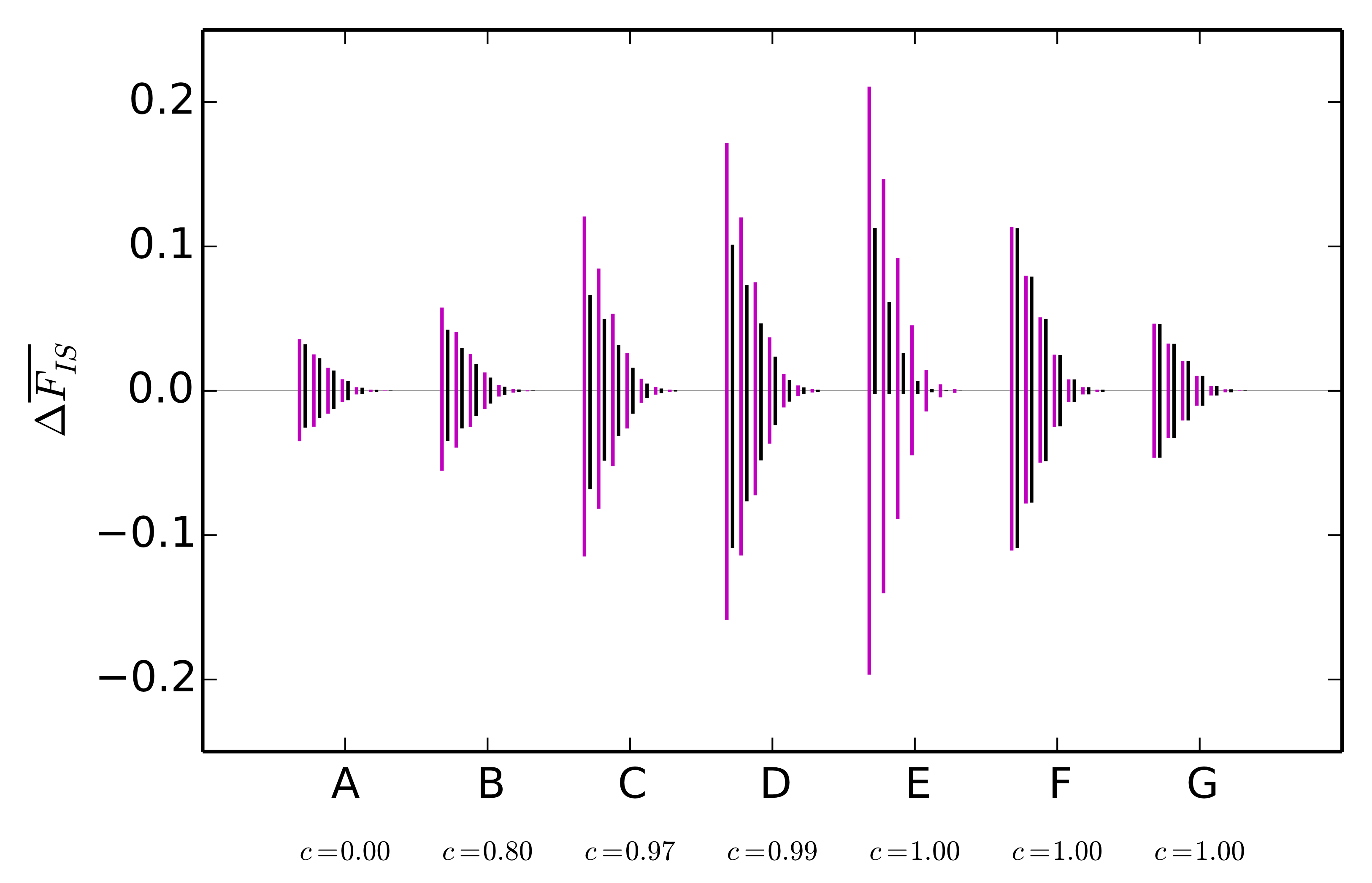
**

**Figure 2.8: Sampling error of the mean** $\bar{\boldsymbol{F}_{\boldsymbol{IS,t,L}}}$ **according to number of loci.** Mean signed deviation $\Delta\bar{F_{\mathrm{IS}}}$ for each parameter set in figure 6 (main manuscript), sampling from the $\tilde{F_{IS,50}}$ distribution at 50 generations after all loci were at $F_{IS,0}=0$ for $\nu_{a}=\nu_{A}$ (left/magenta), or the steady state distribution of $F_{\mathrm{IS}}$ ($\tilde{F_{IS,\infty}}$, right/black). The bars for each parameter set are each based on 105 random samples of 5, 10, 25, 100, 1 000, 10 000 and 100 000 loci (left to right). Model parameters – **A:** $c=0$, $\mu={10}^{-6}$, $N=100$; **B:**$c=0.8$, $\mu={10}^{-6}$, $N=100$; **C:** $c\approx0.97 (t_{c}=t_{N})$, $\mu={10}^{-6}$, $N=100$; **D:** $c=0.99$, $\mu={10}^{-6}$, $N=100$; **E:** $c=1.0$, $\mu={10}^{-6}$, $N=100$; **F:** $c=1.0$, $\mu={10}^{-2}$, $N=100$; **G:** $c=1.0$, $\mu={10}^{-1}$, $N=100.$


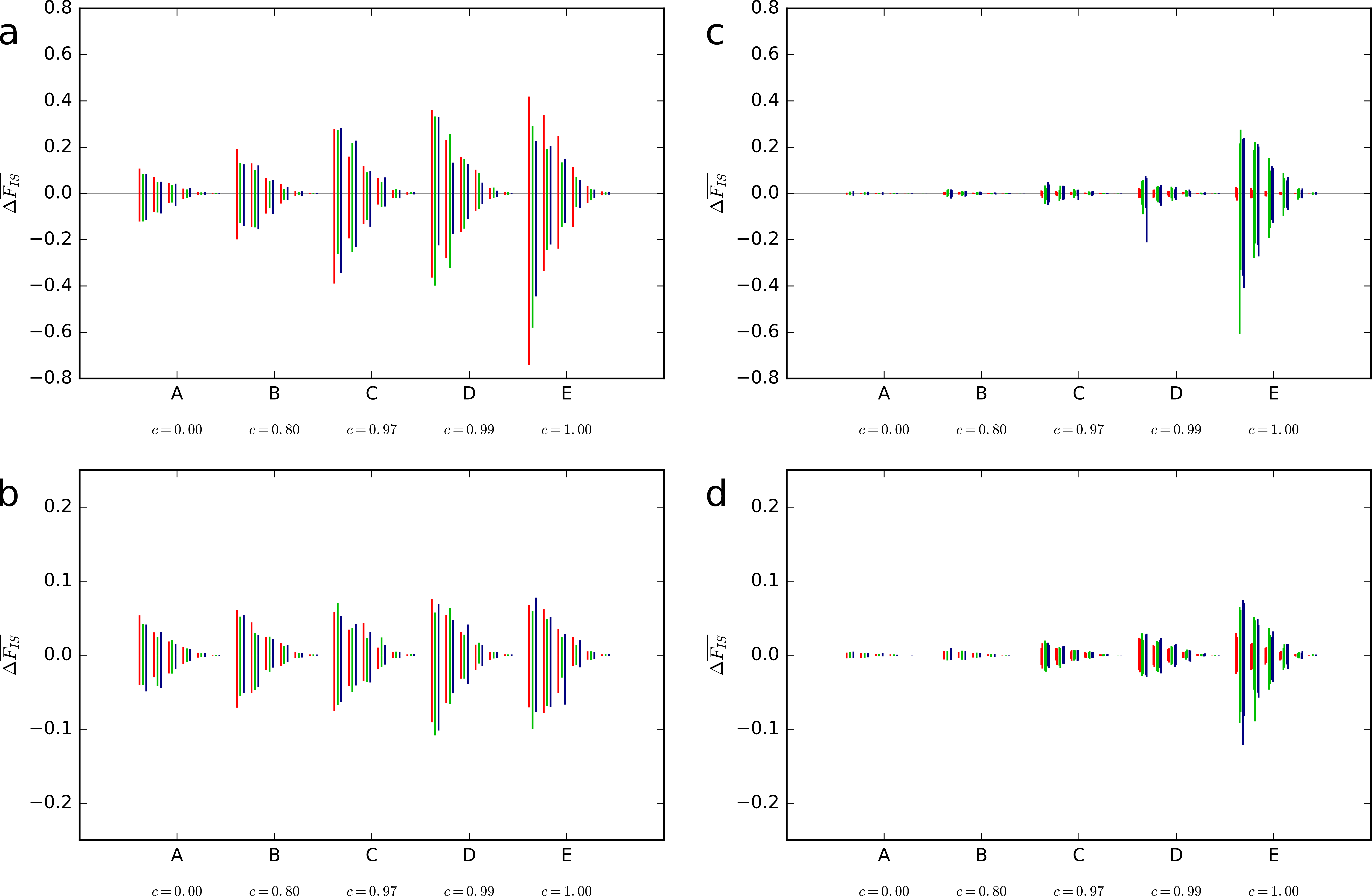


**Figure 2.9: Sampling error of the mean** $\bar{\boldsymbol{F}_{\boldsymbol{IS,t,L}}}$ **according to number of loci.** Mean signed deviation $\Delta\bar{F_{\mathrm{IS}}}$ for four different combinations of population size and mutation rate, sampling from 50 000 example trajectories at 50 (left/red), 10 000 (middle/green) or 90 000 generations (right/blue) after all loci were at $F_{IS,0}=0$ for $\nu_{A1\ldots A10}=1/10$. The bars for each parameter set are each based on 105 random samples of 5, 10, 25, 100, 1 000, 10 000 and 100 000 loci (left to right), results for $N=9 900$ and c ≤ 0.8 from two independent repetitions (double bars). Final $F_{\mathrm{IS}}$ distribution expected at ≥ 90 000 generations (**a**, **c**), ≥ 10 000 generations (**b**) or > 90 000 generations (**d**), respectively. Model parameters – **a:** $\mu={10}^{-1}$, $N=100$; **b:** $\mu={10}^{-3}$, $N=100$; **c:** $\mu={10}^{-3}$, $N=9 900$; **d:** $\mu={10}^{-5}$, $N=9 900$; **A:** $c=0$ **B:**$c=0.8$ **C:** $c=0.97$**D:** $c=0.99$ **E:** $c=1.0$.

**Table 2.1:** Examples for transition probabilities for a biallelic locus (three genotypes *aa, aA, AA*), for different rates of clonality (rows, brown) and mutation rates (columns, black). Current model state $\left( q_{aa}, q_{aA}, q_{AA} \right)_{t}$ in bold blue, next model state $\left( q_{aa}, q_{aA}, q_{AA} \right)_{t+1}$ in bold red, corresponding $F_{IS}$ values (blue/red) in italics.

|  | Rates of clonality | Mutation rates | | | | | |
| --- | --- | --- | --- | --- | --- | --- | --- |
|  |  | 0.0001 | 0.0005 | 0.001 | 0.005 | 0.01 | 0.05 |
|  |  |  |  | **(99,1,0)** | *-0.0050* |  |  |
|  | 0.0 | 0.359727366 | 0.332195838 | 0.300711964 | 0.135339828 | 0.049666529 | 1.35615.10^-5^ |
| **(100,0,0)** | 0.5 | 0.359273551 | 0.331777093 | 0.300333286 | 0.135170775 | 0.049605125 | 1.35461.10^-5^ |
| *n.a.* | 0.8 | 0.359001534 | 0.331526096 | 0.300106307 | 0.135069444 | 0.049568319 | 1.35369.10^-5^ |
|  | 0.99 | 0.358829362 | 0.331367229 | 0.299962641 | 0.135005306 | 0.049545022 | 1.35311.10^-5^ |
|  | 1.0 | 0.358820303 | 0.33135887 | 0.299955081 | 0.135001931 | 0.049543796 | 1.35308.10^-5^ |
|  | 0 | 0.000944895 | 0.001014181 | 0.001092001 | 0.001366965 | 0.001136255 | 4.50585.10^-6^ |
| **(99,0,1)** | 0.5 | 0.000490184 | 0.000594432 | 0.000712198 | 0.001196326 | 0.001073497 | 4.48546.10^-6^ |
| *1.0* | 0.8 | 0.000217902 | 0.000343084 | 0.00048477 | 0.001094145 | 0.001035916 | 4.47324.10^-6^ |
|  | 0.99 | 4.56674.10^-5^ | 0.000184092 | 0.000340908 | 0.001029508 | 0.001012142 | 4.46551.10^-6^ |
|  | 1.0 | 3.6607E.10^-5^ | 0.000175728 | 0.00033334 | 0.001026107 | 0.001010892 | 4.46510.10^-6^ |
|  | 0.0 | 0.368730122 | 0.367100443 | 0.362423852 | 0.272032987 | 0.150244907 | 0.000156341 |
| **(99,1,0)** | 0.5 | 0.369176661 | 0.367479292 | 0.362728906 | 0.272034483 | 0.150183744 | 0.000156196 |
| *-0.0050* | 0.8 | 0.36944377 | 0.36770587 | 0.3629113 | 0.272035177 | 0.150147009 | 0.000156109 |
|  | 0.99 | 0.369612624 | 0.367849086 | 0.363026569 | 0.272035538 | 0.150123729 | 0.000156055 |
|  | 1.0 | 0.369621504 | 0.367856617 | 0.363032631 | 0.272035556 | 0.150122504 | 0.000156052 |
|  | 0.0 | 0.187089303 | 0.200807916 | 0.216216103 | 0.270659152 | 0.224978441 | 0.000892158 |
| **(98,2,0)** | 0.5 | 0.187779608 | 0.201476567 | 0.216853313 | 0.271000637 | 0.225073572 | 0.000891519 |
| *-0.0101* | 0.8 | 0.188193921 | 0.201877819 | 0.217235631 | 0.271205331 | 0.225130525 | 0.000891135 |
|  | 0.99 | 0.188456368 | 0.202131968 | 0.217477761 | 0.271334893 | 0.225166545 | 0.000890892 |
|  | 1.0 | 0.188470182 | 0.202145345 | 0.217490505 | 0.271341711 | 0.22516844 | 0.000890879 |
|  |  |  |  | **(25,50,25)** | *0.0* |  |  |
|  | 0 | 0.008592207 | 0.008592207 | 0.008592207 | 0.008592207 | 0.008592207 | 0.008592207 |
| **(25,49,26)** | 0.5 | 0.008592207 | 0.008592207 | 0.008592207 | 0.008592207 | 0.008592207 | 0.008592207 |
| *0.0199* | 0.8 | 0.008592207 | 0.008592207 | 0.008592207 | 0.008592207 | 0.008592207 | 0.008592207 |
|  | 0.99 | 0.008592207 | 0.008592207 | 0.008592207 | 0.008592207 | 0.008592207 | 0.008592207 |
|  | 1 | 0.008592207 | 0.008592207 | 0.008592207 | 0.008592207 | 0.008592207 | 0.008592207 |
|  | 0 | 0.008760682 | 0.008760682 | 0.008760682 | 0.008760682 | 0.008760682 | 0.008760682 |
| **(25,51,24)** | 0.5 | 0.008760682 | 0.008760682 | 0.008760682 | 0.008760682 | 0.008760682 | 0.008760682 |
| *-0.020* | 0.8 | 0.008760682 | 0.008760682 | 0.008760682 | 0.008760682 | 0.008760682 | 0.008760682 |
|  | 0.99 | 0.008760682 | 0.008760682 | 0.008760682 | 0.008760682 | 0.008760682 | 0.008760682 |
|  | 1 | 0.008760682 | 0.008760682 | 0.008760682 | 0.008760682 | 0.008760682 | 0.008760682 |
|  |  |  |  | **(25,51,24)** | -0.020 |  |  |
|  | 0 | 0.008338326 | 0.008338593 | 0.008338926 | 0.008341587 | 0.0083449 | 0.008370845 |
| **(25,49,26)** | 0.5 | 0.008128797 | 0.008129457 | 0.00813028 | 0.008136834 | 0.008144944 | 0.008206627 |
| *0.0199* | 0.8 | 0.007966865 | 0.00796787 | 0.007969124 | 0.007979096 | 0.007991406 | 0.008083858 |
|  | 0.99 | 0.007851178 | 0.007852439 | 0.007854013 | 0.007866524 | 0.00788195 | 0.007997164 |
|  | 1 | 0.007844818 | 0.007846094 | 0.007847686 | 0.007860337 | 0.007875936 | 0.007992414 |
|  | 0 | 0.008847013 | 0.008847154 | 0.008847331 | 0.008848738 | 0.008850482 | 0.008863804 |
| **(20,50,25)** | 0.5 | 0.008801569 | 0.008801855 | 0.008802211 | 0.008805038 | 0.008808509 | 0.008833886 |
| *0.0* | 0.8 | 0.008731975 | 0.008732481 | 0.008733111 | 0.008738098 | 0.008744199 | 0.008787966 |
|  | 0.99 | 0.008671851 | 0.008672545 | 0.008673409 | 0.00868025 | 0.008688608 | 0.008748196 |
|  | 1 | 0.008668348 | 0.008669052 | 0.00866993 | 0.008676878 | 0.008685368 | 0.008745876 |
|  | 0 | 0.00884873 | 0.008848729 | 0.008848729 | 0.008848721 | 0.008848694 | 0.008847845 |
| **(25,51,24)** | 0.5 | 0.008983807 | 0.008983661 | 0.008983479 | 0.00898201 | 0.008980141 | 0.008964072 |
| *-0.020* | 0.8 | 0.009022025 | 0.009021931 | 0.009021813 | 0.009020832 | 0.009019518 | 0.009005848 |
|  | 0.99 | 0.009029325 | 0.009029319 | 0.009029309 | 0.009029175 | 0.009028866 | 0.009021267 |
|  | 1 | 0.009029345 | 0.009029344 | 0.009029342 | 0.009029264 | 0.009029022 | 0.00902184 |
|  | 0 | 0.008333036 | 0.008332903 | 0.008332736 | 0.008331394 | 0.008329703 | 0.008315587 |
| **(25,52,23)** | 0.5 | 0.008633736 | 0.008633176 | 0.008632477 | 0.008626882 | 0.008619896 | 0.008564401 |
| *-0.040* | 0.8 | 0.008776748 | 0.008776058 | 0.008775194 | 0.008768279 | 0.008759609 | 0.008689582 |
|  | 0.99 | 0.008851907 | 0.008851187 | 0.008850286 | 0.008843048 | 0.008833937 | 0.008759002 |
|  | 1 | 0.008855524 | 0.008854803 | 0.008853901 | 0.008846658 | 0.008837538 | 0.008762439 |
| **Table 2.1** *continued* | | | | | | | |
|  | Rates of clonality | Mutation rates | | | | | |
|  |  | 0.0001 | 0.0005 | 0.001 | 0.005 | 0.01 | 0.05 |
|  |  |  |  | **(1,99,0)** | *-0.9802* |  |  |
|  | 0 | 1.00594.10^-27^ | 1.00593.10^-27^ | 1.00591.10^-27^ | 1.00574.10^-27^ | 1.00554.10^-27^ | 1.00384.10^-27^ |
| **(2,98,0)** | 0.5 | 2.53351.10^-11^ | 2.41318.10^-11^ | 2.27080.10^-11^ | 1.39665.10^-11^ | 7.61613.10^-12^ | 6.32255.10^-14^ |
| *-0.9608* | 0.8 | 0.00023191 | 0.000218742 | 0.000203312 | 0.000112909 | 5.37668.10^-5^ | 1.20573.10^-7^ |
|  | 0.99 | 0.175509262 | 0.172560875 | 0.168517811 | 0.128511391 | 0.0795377 | 0.000252415 |
|  | 1 | 0.184846809 | 0.184433629 | 0.18320146 | 0.154025233 | 0.102088939 | 0.000363639 |
|  | 0 | 3.98395.10^-29^ | 3.98395.10^-29^ | 3.98395.10^-29^ | 3.98394.10^-29^ | 3.98393.10^-29^ | 3.98355.10^-29^ |
| **(1,99,0)** | 0.5 | 2.87599.10^-12^ | 2.73400.10^-12^ | 2.56637.10^-12^ | 1.54797.10^-12^ | 8.24185.10^-13^ | 5.74487.10^-15^ |
| *-0.9802* | 0.8 | 7.07195.10^-5^ | 6.63099.10^-5^ | 6.11805.10^-5^ | 3.20868.10^-5^ | 1.42803.10^-5^ | 2.07982.10^-8^ |
|  | 0.99 | 0.278339668 | 0.265330635 | 0.249605053 | 0.147004377 | 0.070689923 | 7.84586.10^-5^ |
|  | 1 | 0.366069914 | 0.351464864 | 0.333373667 | 0.205737631 | 0.102120188 | 0.000117553 |
|  | 0 | 3.82776.10^-29^ | 3.82788.10^-29^ | 3.82803.10^-29^ | 3.82926.10^-29^ | 3.83078.10^-29^ | 3.84269.10^-29^ |
| **(0,99,1)** | 0.5 | 2.65908.10^-12^ | 2.52826.10^-12^ | 2.37379.10^-12^ | 1.43437.10^-12^ | 7.65342.10^-13^ | 5.41191.10^-15^ |
| *-0.9802* | 0.8 | 5.87522.10^-5^ | 5.51562.10^-5^ | 5.09664.10^-5^ | 2.70330.10^-5^ | 1.21811.10^-5^ | 1.88432.10^-8^ |
|  | 0.99 | 0.056533332 | 0.060332095 | 0.063836578 | 0.062523958 | 0.03913655 | 6.62913.10^-5^ |
|  | 1 | 0.003588924 | 0.016584937 | 0.030058585 | 0.068352548 | 0.051065304 | 9.86775.10^-5^ |
|  | 0 | 7.81014.10^-31^ | 7.81027.10^-31^ | 7.81042.10^-31^ | 7.81167.10^-31^ | 7.81321.10^-31^ | 7.82497.10^-31^ |
| **(0,100,0)** | 0.5 | 1.61606.10^-13^ | 1.53325.10^-13^ | 1.43570.10^-13^ | 8.49259.10^-14^ | 4.41489.10^-14^ | 2.58388.10^-16^ |
| *-1.0* | 0.8 | 1.06749.10^-5^ | 9.95016.10^-6^ | 9.11311.10^-6^ | 4.51368.10^-6^ | 1.87744.10^-6^ | 1.77586.10^-9^ |
|  | 0.99 | 0.218501976 | 0.201947116 | 0.183006341 | 0.083238473 | 0.031099054 | 1.20718.10^-5^ |
|  | 1 | 0.358856912 | 0.331534644 | 0.300288605 | 0.136031909 | 0.050564966 | 1.88106.10^-5^ |

**Table 2.2:** Convergence time of genetic drift $t_{N}$ based on numerical solutions for the Markov chain absorption time. Results for different population sizes $N$, numbers of alleles $n$ and resulting numbers of genotypes $g$.

| $n=2, g=3$ | | | | $n=3, g=6$ | | $n=4, g=10$ | |
| --- | --- | --- | --- | --- | --- | --- | --- |
| $N$ | $t_{N}$ | $N$ | $t_{N}$ | $N$ | $t_{N}$ | $N$ | $t_{N}$ |
| $2$ | $2.0$ | $20$ | $30.8$ | $2$ | $2.0$ | $2$ | $2.0$ |
| $3$ | $3.9$ | $30$ | $46.9$ | $3$ | $3.9$ | $3$ | $3.9$ |
| $4$ | $5.2$ | $40$ | $63.0$ | $4$ | $5.8$ | $4$ | $5.8$ |
| $5$ | $6.8$ | $50$ | $79.2$ | $5$ | $7.7$ | $5$ | $7.7$ |
| $6$ | $8.5$ | $60$ | $95.4$ | $6$ | $9.7$ | $6$ | $9.7$ |
| $7$ | $10.0$ | $70$ | $111.5$ | $7$ | $11.3$ | $7$ | $11.6$ |
| $8$ | $11.6$ | $80$ | $127.7$ | $8$ | $13.0$ | $8$ | $13.6$ |
| $9$ | $13.3$ | $90$ | $143.9$ | $9$ | $14.8$ | $-$ | $-$ |
| $10$ | $14.8$ | $100$ | $160.1$ | $10$ | $16.6$ | $-$ | $-$ |
| $15$ | $22.8$ | $120$ | $192.4$ | $15$ | $25.7$ | $-$ | $-$ |

**Table 2.3:** Convergence time of genetic drift $t_{N}$ based on simulations. For each of 10 000 randomly chosen start states, the maximum time to genotypic uniformity was obtained from 10 000 chains of multinomial sampling. The $t_{N}$ values represent the mean over these maximal times for all sampled start states. Results for different population sizes $N$ and numbers of alleles $n$.

| $N$ | $n$ | | | |
| --- | --- | --- | --- | --- |
|  | **2** | **4** | **10** | **50** |
| **2** | 2.0 | 2.0 | 2.0 | 2.0 |
| **5** | 5.5 | 5.7 | 5.7 | 5.7 |
| **10** | 11.5 | 12.1 | 11.8 | 12.0 |
| **50** | 60.0 | 63.3 | 62.2 | 63.4 |
| **100** | 119.6 | 126.6 | 127.4 | 126.6 |
| **500** | 594.7 | 648.4 | 631.9 | 645.8 |
| **1000** | 1202.3 | 1288.6 | 1295.9 | 1299.6 |
| **5000** | 5983.4 | 6446.1 | 6520.0 | 6430.8 |
| **10000** | 12214.5 | 12826.8 | 13116.0 | 12995.2 |
| **50000** | 59957.6 | 65917.4 | 64939.0 | 64940.5 |
| **100000** | 121959.8 | 128884.3 | 126964.5 | 129512.3 |
| **500000** | 607228.7 | 650342.5 | 646640.0 | 656408.9 |

**Table 2.4:** Effects of different rates of partial clonality on the dynamics of $F_{IS}$. Text in italics refers to figure 2.7 (additional file 2.6).

| **Allele fixation rate** | *Right plot lines* | Identical for all start $F_{IS}$ | Slight dependence on start $F_{IS}$ | Pronounced dependence on start $F_{IS}$ | $F_{IS}=-1$ hardly fixes, $F_{IS}=0$ at reduced rate, $F_{IS}=1$ very fast | Depends on $\mu$ & $N$ |
| --- | --- | --- | --- | --- | --- | --- |
| $\mathbf{F}_{\mathbf{IS}}$ **values  after ten generations** | *Left plot dots* | independent of start $F_{IS}$ | slightly biased according to  start $F_{IS}$ | distinct according to  start $F_{IS}$ | distinct according to  start $F_{IS}$ | Depends on $\mu$ |
| **Alternation of positive and negative** $\mathbf{F}_{\mathbf{IS}}$ | *Central plot thin lines* | High frequency | Low frequency | Very low frequency | Rare except for recent loss of sex or near fixation | Depends on $\mu$ & $N$ |
| **Variation of** $\mathbf{F}_{\mathbf{IS}}$ **values** | *Central plot grey area / thin lines, left plot thin lines* | Depends on $N$ only | Increased | Full range | Depends on start $F_{IS}$ | Depends on $N$ |
| **Convergence speed** | *Central plot thick lines  and* $t_{c}$ | One/very few generations | Several generations | Many generations | Depends on $N$ | Depends on $\mu$ |
| **Convergence to a common mean** $\mathbf{F}_{\mathbf{IS}}$ | *Central plot thick lines* | Yes, $F_{IS}=0$ | Yes, $F_{IS}\approx0$ | Yes, $F_{IS}<0$ | No, fixation or $F_{IS}=-1$ | Yes, $F_{IS}=0$ |
| ***Fig.2.7*** | *Where to look* | *Row A* | *Row B* | *Row C* | *Row D* | *Row E* |
|  | **Rate of  clonal reproduction** | Exclusively sexual to intermediate rate | Intermediate to high rate | High to very high rate | Genetic drift dominates ($N$ small) | Mutation dominates ($N$ very big) |
|  |  |  |  |  | Very high rate to exclusively clonal | |
