# Supplementary material for: Rare sex or out of reach equilibrium? The dynamics of FIS in partially clonal organisms
Source: BMC Genet. 2016 Jun 10;17:76. doi: 10.1186/s12863-016-0388-z (PMC4902967; doi:10.1186/s12863-016-0388-z)
Supplement: Additional file 4: — Literature review. References and supplementary data for the literature review. “PN” refers to the number of the dataset(s) in Fig. 5, *: F IS was calculated from H e and H o. (DOCX 20 kb) [file 12863_2016_388_MOESM4_ESM.docx]

**Additional file 4: Literature review**

**References used in the literature review**

We conducted a Web of Science search with the exact search term [(microsatellite OR "SSR" OR "simple sequence repeat" or "SNP" or "single nucleotide polymorphism") AND (clonal OR asexual OR vegetative OR apomictic OR apomixis OR agamospermy OR parthenogenesis)], which yielded 5480 references. Screening the 2000 most recent references yielded 21 studies that were accessible and reported relevant data ($F_{IS}$ values; no known population substructure, no filtering of repeated MLGs, no cyclical parthenogenesis, dominantly diploid life cycle). Six of these studies reported only mean $F_{IS}$ values over all loci and two were of species combining selfing and asexuality. The remaining 13 are listed below.

| Reference | Details | PN |
| --- | --- | --- |
| S. Duran, M. Pascual, A. Estoup, X. Turon (2004): Strong population structure in the marine sponge *Crambe crambe* (Poecilosclerida) as revealed by microsatellite markers | *Crambe crambe*, Porifera  Mediterranean + Atlantic | 1-11 |
| T. Nagamitsu, M. Ogawa, K. Ishida, H. Tanouchi (2004): Clonal diversity, genetic structure, and mode of recruitment in a *Prunus ssiori* population established after volcanic eruptions | *Prunus ssiori*, Angiospermae  Japan | 13 |
| S. Stoeckel, J. Grange, J. Fernandez-Manjarres, I. Bilger, N. Frascaria-Lacoste, S. Mariette (2006): Heterozygote excess in a self-incompatible and partially clonal forest tree species – *Prunus avium* L. | *Prunus avium*, Angiospermae  France | 14-16 |
| J. M. Corral, M. Puente Molins, O. M. Aliyu, T. F. Sharbel (2011): Isolation and characterization of microsatellite loci from apomictic *Hypericum perforatum* (Hypericaceae) | *Hypericum perforatum*, Angiospermae  USA, Czech Republic, Germany | 17*-20* |
| K. Jiang, H. Gao, N.-N. Xu, E. P. Keung Tsang, X. Chen (2011): A set of microsatellite primers for *Zostera japonica* (Zosteraceae) | *Zostera japonica*, Angiospermae  China, Taiwan | 21*-22* |
| J. M. Tew, S. L. Lance, K. L. Jones, S. D. Fehlberg (2012): Microsatellite development for an endangered riparian inhabitant, *Lilaeopsis schaffneriana* subsp. *recurva* (Apiaceae) | *Lilaeopsis schaffneriana* ssp. *recurva*, Angiospermae  USA, Mexico | 23*-24* |
| W. Liu, Y. Zhou, H. Liao, Y. Zhao, Z. Song (2011): Microsatellite primers in *Carex moorcroftii* (Cyperaceae), a dominant species of the steppe on the Qinghai-Tibetan Plateau | *Carex moorcroftii*, Angiospermae  China | 25*-28* |
| C. Barnabe, R. Buitrago, P. Bremond, C. Aliaga, R. Salas, P. Vidaurre, C. Herrera, F. Cerqueira, M.-F. Bosseno, E. Waleckx, S. F. Breniere (2013): Putative panmixia in restricted populations of *Trypanosoma cruzi* isolated from wild *Triatoma infestans* in Bolivia | *Trypanosoma cruzi*, Euglenozoa  Bolivia | 30-35 |
| S. W. M. Tesson, M. Borra, W. H. C. F. Kooistra, G. Procaccini (2011): Microsatellite primers in the planktonic diatom *Pseudo-nitzschia multistriata* (Bacillariophyceae) | *Pseudo-nitzschia multistriata*, Heterokonta  Italy | 36*-39* |
| L. Villate, D. Esmenjaud, M. van Helden, S. Stoeckel, O. Plantard (2010): Genetic signature of amphimixis allows for the detection and fine scale localization of sexual reproduction events in a mainly parthenogenetic nematode | *Xiphinema index*, Nematoda  France | 40-45 |
| H. Gao, K. Jiang, Y. Geng, X.-Y. Chen (2012): Development of microsatellite primers of the largest seagrass, *Enhalus acoroides* (Hydrocharitaceae) | *Enhalus acoroides*, Angiospermae  China | 46*-47* |
| L. M. McInnes, A. P. Dargantes, U .M. Ryan, S. A. Reid (2012): Microsatellite typing and population structuring of *Trypanosoma evansi* in Mindanao, Philippines | *Trypanosoma evansi*, Euglenozoa  Philippines | 48-49 |
| R. Vilas, A. Cao, B. G. Pardo, S. Fernández, A. Villalba, P. Martínez (2011): Very low microsatellite polymorphism and large heterozygote deficits suggest founder effects and cryptic structure in the parasite *Perkinsus olseni* | *Perkinsus olseni*, Alveolata  Spain | 50-53 |

**Table 3.1:** References and supplementary data for the literature review. “PN” refers to the number of the dataset(s) in figure 9, *: $F_{IS}$ was calculated from $H_{e}$ and $H_{o}$.
